# Mendelian Randomization Uncovers Potential Repurposable Medications for Neuropsychiatric Disorders

**DOI:** 10.2174/011570159X368382250527073353

**Published:** 2025-06-10

**Authors:** Xiao Xiao, Tingyu Li, Qiang Wang, Linbo Gao, Shanling Liu, Lin Zhang

**Affiliations:** 1 Department of Medical Genetics, West China Second University Hospital, Sichuan University, Chengdu, Sichuan, China;; 2 Key Laboratory of Birth Defects and Related Diseases of Women and Children (Sichuan University), Ministry of Education, Chengdu, Sichuan, China;; 3 Laboratory of Molecular Translational Medicine, Center for Translational Medicine, Key Laboratory of Birth Defects and Related Diseases of Women and Children (Sichuan University), Ministry of Education, West China Second University Hospital, Sichuan University, Chengdu, Sichuan, People’s Republic of China

**Keywords:** Drug repurposing, causal relationships, mendelian randomization, genetic associations, neuropsychiatric disorders, traditional pharmacotherapies

## Abstract

**Background:**

The growing prevalence of neuropsychiatric disorders is becoming a major health challenge. Traditional pharmacotherapies face limitations, making drug repurposing a valuable strategy. However, high-throughput screening approaches for these conditions are scarce.

**Methods:**

This study leveraged exposure data from the UK Biobank Neale Lab (N = 361,141) and outcome data from the FinnGen database (N = approximately 410,000) to employ Mendelian Randomization (MR) analyses and identify potential drug repurposing candidates for neuropsychiatric disorders. Sensitivity, Linkage Disequilibrium Score Correlation (LDSC), and Bayesian Colocalization (COLOC) analyses were conducted to ensure the robustness and reliability of our findings.

**Results:**

Using the IVW method, seven medications with negative causal associations with neuropsychiatric disorders were identified. Pregabalin, bumetanide, and prednisolone were associated with reduced anxiety (beta = -7.28, *p* = 4.00e-03; beta = -2.24, *p* = 6.00e-03; beta = -1.74, *p* = 2.84e-03). Vitamin B_1_ preparations showed an inverse association with dementia (beta = -2.47, *p* = 1.51e-03), Creon E/C granules with epilepsy (beta = -4.99, *p* = 3.91e-03), Pentasa SR 250 mg with multiple sclerosis (beta = -3.95, *p* = 3.83e-03), and zolmitriptan with stroke excluding subarachnoid hemorrhage (beta = -1.61, *p* = 6.00e-03). Sensitivity analyses confirmed these findings, whereas the LDSC and COLOC analyses provided additional support.

**Conclusion:**

MR-based drug repurposing is a promising approach for the treatment of neuropsychiatric disorders. Further validation is necessary to effectively integrate these medications into clinical practice.

## 1 INTRODUCTION

Neuropsychiatric disorders involve multiple pathological mechanisms, including neuronal damage, loss of function, loss of synaptic connectivity, and cell death. With the increasing complexity of environmental factors, rising social pressures, and a growing aging population, the prevalence of neuropsychiatric disorders, such as Alzheimer's disease (AD), Parkinson's disease (PD), and autism spectrum disorder, has been steadily increasing. As of 2021, approximately 3.4 billion individuals have been affected by neurological issues, attributed to 11.1 million deaths. These disorders are the leading causes of disability-adjusted life years (DALYs) and years of life lost (YLL), imposing a significant burden on global health [1]. In the United States, over one in nine individuals aged 65 years (10.9%) were diagnosed with AD. It is predicted that the number of cases will reach 13.8 million by 2060, representing over 48% of the elderly population aged 65 and above, which will not only bring a huge burden to the patient’s family but also have a serious impact on the economy [2]. In 2019, the global economic cost of AD was estimated at $1.3 trillion, and it is expected to be $2.8 trillion by 2030 [3].

Pharmacotherapy is effective in treating neuropsychiatric disorders, but it faces several challenges. Symptom overlap increases the complexity of diagnosis and treatment, leading to difficulties in drug use. Some medications can cause severe side effects resulting in poor patient tolerance. Additionally, developing new drugs is a lengthy process that requires substantial financial investments. Prescribing medications that are already on the market allows healthcare providers to take advantage of their well-established safety profiles and known side effects, which have been thoroughly assessed through preclinical studies, clinical trials, and long-term follow-up. This practice, known as drug repurposing, involves exploring new uses for these established drugs and has the potential to lead to effective treatments with predictable side effects, ultimately helping to reduce healthcare costs [4-6]. For instance, colchicine, traditionally used for gout, is currently being studied for its ability to reduce inflammation related to COVID-19 [7, 8]. Similarly, metformin, which is primarily prescribed for diabetes, is now being investigated for its possible anti-cancer effects [9]. Thalidomide, once infamous for causing birth defects, has been repurposed for the treatment of multiple myeloma [10]. Additionally, auranofin, originally developed as an anti-rheumatic drug, is being researched for its effectiveness against gastrointestinal stromal tumors [11]. These examples highlight the significant benefits of drug repurposing for broadening treatment options and improving the efficiency of healthcare systems.

Mendelian Randomization (MR) is a statistical approach based on Mendel’s laws of random inheritance used to infer causal associations between exposure and outcome [12]. This approach effectively avoids reverse causation and provides evidence comparable to that of randomized controlled trials [13]. Previously, Zhang *et al.* utilized this methodology to identify cannabidiol, doxorubicin, genistein, and propylthiouracil as potential treatments for manic episodes and type 1 diabetes [14]. Duan *et al.* found that fostamatinib, amlexanox, BIIB-023, RG-7212, and glutathione may be effective in treating Amyotrophic Lateral Sclerosis (ALS), and cell experiments confirmed that fostamatinib and amlexanox can reduce neuroinflammation in ALS models [15]. These examples reveal the significant potential of the MR methodology in drug screening and repurposing. Linkage Disequilibrium Score Correlation (LDSC), used to estimate genetic correlations between phenotypes, and Bayesian Colocalization (COLOC), employed to determine whether phenotypes share one genetic basis, both of which provide crucial support to MR results, ensuring that discovered causal associations are more likely to be genuine rather than confounded by other factors [16, 17].

542 treatments/medications were collected as exposures from the UK Biobank Neale Lab and 15 neuropsychiatric diseases from the FinnGen database as outcomes for the MR analysis [18, 19]. The robustness of the results was ensured through sensitivity tests, and further exploration of genetic associations was conducted using LDSC and COLOC analyses. Ultimately, we identified medications that may exert negative effects on neuropsychiatric diseases, thereby offering new insights into drug repurposing.

## 2 METHODS

### 2.1 Study Design

This study employed an MR analysis utilizing exposure data from the UK Biobank Neale Lab Round 2 (http://www.nealelab.is/uk-biobank/), encompassing a variety of conventional treatments and medications. The outcomes of interest consisted of 15 neurological and psychiatric disorders sourced from the FinnGen database. The flowchart of the study design is shown in Fig. (**1**). In brief, our MR analysis was based on three key assumptions: (1) Instrumental variants (IVs) were strongly associated with exposure; (2) IVs were independent of confounding factors; and (3) IVs affected the outcome only through exposure, that is, IVs were not directly associated with the outcome. This study focused on the results with statistical significance, which showed negative associations indicative of a therapeutic effect. Sensitivity analyses were used to assess pleiotropy and heterogeneity using leave-one-out tests to confirm potential treatments. The validated candidates were further scrutinized using LDSC and COLOC for genetic association exploration.

### 2.2 Data Source for Exposure/Outcome

The exposure data were sourced from a comprehensive genome-wide association study (GWAS) within the UK Biobank, relying on interview-derived prescription information. Specifically, the data on medication use included 542 GWAS datasets for treatment and medication across both sexes, encompassing 361,141 unrelated individuals of European ancestry. These datasets cover a diverse range of categories, including cardiovascular, respiratory, digestive, neurological, endocrine, metabolic, skin, immunological, and rheumatic disorders, as well as infections, inflammation, supplements, adjuvant therapies, various symptoms, and nonspecific health issues (Table **S1**). Neuropsychiatric disorder data, serving as the outcome, were derived from 15 GWAS datasets obtained from the FinnGen database. These diseases included attention deficit hyperactivity disorder (ADHD), all anxiety disorders, anorexia nervosa, bipolar affective disorders, dementia, obsessive-compulsive disorder, schizophrenia, AD, epilepsy, migraine, multiple sclerosis (MS), PD, stroke (excluding subarachnoid hemorrhage, SAH), autism, and substance abuse. Detailed information on both datasets is provided in Table **1**.

### 2.3 Selection of IVs

A two-sample MR was used to explore the causal relationship between treatments/medications and neuropsychiatric disorders. Following the STROBE-MR checklist and the guidelines of Haycock *et al.*, we identified IVs that met essential MR assumptions [20, 21]. Genome-wide significant SNPs (Table **S2**) were assessed within a 10,000 kb window and an r^2^ < 0.001 threshold to handle linkage disequilibrium. Palindromic SNPs, those linked to outcomes at *p* < 0.01, and SNPs absent in the outcomes were excluded. Confounding factors, specifically hypertension, diabetes, smoking, alcohol consumption, and sleep disorders, were controlled by excluding the related SNPs identified in PhenoScanner V2 [22]. All the selected SNPs had *F*-statistics > 10, guaranteeing strong instrumental validity [23].

### 2.4 MR Analysis and Sensitivity Test

Five MR methods were used in this study [24]. The primary method employed is the Inverse Variance Weighted (IVW) method. It combines estimates from genetic variants weighted by inverse variances, assuming that they meet the key assumptions for causal effect estimation. To ensure analytical robustness, this study used four other methods as supplementary approaches. The weighted median method is robust when some variants do not fully meet these assumptions. The MR-Egger regression detects horizontal pleiotropy. The simple mode determines the causal effect direction based on the majority of variant effects. The weighted mode considers the effect magnitudes to obtain a more accurate combined effect. Using these methods, we aimed to obtain reliable and comprehensive insights.

A series of sensitivity analyses were conducted to ensure the robustness of the MR results [24]. For the heterogeneity analysis, Cochran's Q test was utilized to identify SNP effect heterogeneity [25]. The pleiotropy test, commonly associated with the MR-Egger intercept, was employed to ascertain whether the IVs exerted a direct influence on the outcome through pathways distinct from the exposure [26]. The threshold for both tests was set at *p* > 0.05. The leave-one-out method typically involves systematically removing one variable at a time to determine whether a specific SNP has an independent effect on the MR outcomes. In this study, SNPs that were found to influence the results independently were excluded.

### 2.5 LDSC and COLOC Analyses

To complement the MR results, LDSC was used to estimate the genetic correlation between exposure and outcome [17]. This method helps identify a shared genetic architecture and supports the presence of a causal relationship. The genetic correlation coefficient (Rg) from LDSC indicates the nature of this correlation: Rg < 0 suggests a negative correlation and *vice versa*. Additionally, COLOC was employed to assess the probability that the same causal variant underlies both traits [27]. This approach enhances the robustness of our findings by reducing the likelihood of horizontal pleiotropy and provides additional evidence for the proposed causal pathway. COLOC presents five hypotheses: H0 states that there are no shared causal variants; H1 claims that one phenotype has a causal variant while the other does not; H2 asserts that each phenotype has its causal variants; H3 suggests that the causal variants of each phenotype affect one another; and H4 proposes that both phenotypes share a common causal variant. A non-significant posterior probability of H4 (PPH4) supports the exclusive effect of IVs on the outcome *via* exposure, aligning with MR's exclusion restriction.

### 2.6 Statistical Analysis

Given the exploratory nature of this study, a *p*-value threshold of < 0.05 was adopted to determine the statistical significance of our MR analyses. For the LDSC and COLOC analyses, a *p*-value threshold of < 0.05 was used in sensitivity analysis to indicate that heterogeneity and horizontal pleiotropy were not statistically significant, thereby supporting the robustness of the results.

All MR analyses were performed by the TwoSampleMR R package (version 4.3.1, https://mrcieu.github.io/TwoSampleMR/). LDSC analysis was performed using the ldscr R package (version 0.1.0, https://github.com/mglev1n/ldscr/). COLOC analysis was performed using the Coloc R package (version. 5.2.3; https://github.com/chr1swallace/coloc/).

## 3 RESULTS

### 3.1 Selection of IVs

To satisfy the relevance assumption, this study screened the IVs using a threshold of 5e-8. For cases with two or fewer IVs, the threshold was adjusted to 5e-6. Among the 542 treatment/medication codes, 450 utilized the 5e-8 threshold, whereas 92 employed the 5e-6 threshold (Table **S1**). Upon validation, the selected IVs exhibited *F*-statistics exceeding 10, indicating that these instruments possessed adequate strength.

### 3.2 Identification of Treatment/Medication with Negative Causal Associations to Neuropsychiatric Disorders

Using the IVW method, the treatments and medications were identified as associated with a reduced risk of neuropsychiatric disorders. Notably, we observed negative causal associations between pregabalin, bumetanide, and prednisolone and anxiety disorders (beta = -7.28, *p* = 4.00e-03; beta = -2.24, *p* = 6.00e-03; beta = -1.74, *p* = 2.84e-03, respectively). Additionally, vitamin B1 preparations were negatively associated with dementia (beta = -2.47, *p* = 1.51e-03), creon E/C granules in capsules with epilepsy (beta = -4.99, *p* = 3.91e-03), pentasa SR 250 mg modified-release tablets with multiple sclerosis (beta = -3.95, *p* = 3.83e-03), and zolmitriptan with stroke, excluding subarachnoid hemorrhage (beta = -1.61, *p* = 6.00e-03). Results from other MR methods, including MR-Egger, weighted median, simple mode, and weighted mode, consistently indicated a negative correlation between exposure and outcome. The forest plot is shown in Fig. (**2**), and the scatter plot is shown in Fig. (**S1**).

All eligible results underwent sensitivity analysis, which showed that heterogeneity and horizontal pleiotropy were not statistically significant (Table **2**). Additionally, leave-one-out analysis indicated that no single SNP significantly influenced the results (Fig. **S1**). Ultimately, we identified seven significant pairs.

### 3.3 Genetic Correlation and Colocalization Results

To further assess the genetic associations between treatment/medication and neuropsychiatric disorders, the LDSC method was employed. Except for the genetic correlation between zolmitriptan and stroke (excluding SAH) (Rg = -0.549, *p* = 0.049), all other pairs showed no statistically significant results. Notably, the genetic correlations between bumetanide and all anxiety disorders, as well as between creon e/c granules and epilepsy, could not be calculated because of negative heritability estimates (Table **S3**). COLOC analyses were conducted to explore whether the selected medications and their corresponding neuropsychiatric disorders shared common genetic components. None of the results reached the threshold for PPH4, suggesting limited confounding factors and reinforcing the exclusionary assumption of MR (Table **S3**, Fig. **S2**).

## 4 DISCUSSION

In this study, the causal associations were examined between 542 treatments/medications and 15 neuropsychiatric disorders and identified seven medications (pregabalin, bumetanide, prednisolone, vitamin B1 preparation, creon e/c granules, pentasa SR 250 mg m/r tablet, and zolmitriptan) with significant causal relationships to neuropsychiatric disorders. Of these, the first three drugs showed causality with all anxiety disorders, whereas the remaining four drugs were causally linked to dementia, epilepsy, multiple sclerosis, and stroke (excluding SAH). These findings provide evidence for the potential application of these drugs for the prevention and treatment of neuropsychiatric disorders.

Pregabalin, a potential drug for treating neuropsychiatric disorders, has been approved by the Food and Drug Administration for the treatment of diabetic peripheral neuropathy, spinal cord injury, post-herpetic neuralgia, and fibromyalgia [28-31]. Selective Serotonin Reuptake Inhibitors (SSRIs) and Serotonin-Norepinephrine Reuptake Inhibitors (SNRIs) are the first-line medications for anxiety, while pregabalin has a stronger sedative effect than them [32]. Using this drug may improve sleep disorders in anxiety patients earlier than using SSRIs or SNRIs [33]. Additionally, pregabalin is not affected by liver metabolism, has less impact on combination medications, and poses a lower risk of adverse reactions, effectively enhancing the safety of drug use [34]. In a randomized, double-blind, placebo-controlled trial for Generalized Anxiety Disorder (GAD), pregabalin 200 mg three times daily (600 mg/day) showed significant efficacy *versus* placebo by week 1 with good tolerability [35]. In a sequential open-label/double-blind study with 153 generalized social anxiety disorder (GSAD) patients, responders to 10-week open-label pregabalin (450 mg/day) were randomized to 26-week continuation (same dose) or placebo. The results further suggest the therapeutic effect of pregabalin on GSAD [35, 36]. Pregabalin significantly delayed relapse, maintained symptom improvement, and was well-tolerated, supporting its effectiveness for both short- and long-term treatment of anxiety disorders [37]. Our MR analysis further extended the potential application of pregabalin to all anxiety disorders. The rationale of this study is further supported by the mechanism of action of pregabalin, which involves binding to the α2δ subunit of voltage-gated calcium channels, leading to reduced calcium influx, decreased neurotransmitter release, and attenuated excitability of the central nervous system [38, 39].

Bumetanide, a loop diuretic, has been shown in previous studies to alleviate anxiety symptoms by modulating GABA(A) receptor signaling [40]. Since loop diuretics are antagonists of NKCC1 and KCC2 cotransporters, Krystal *et al.* indicated that the cation-chloride cotransport system may be a potential mechanism underlying the anxiolytic effect of bumetanide [41]. In the Fmr1 knockout mouse model, which exhibits sensory hypersensitivity akin to fragile X syndrome, bumetanide treatment normalizes cortical circuit dysfunction and alleviates anxiety-related behaviors by modulating the activity of layer 2/3 pyramidal neurons and enhancing feedforward inhibition in the somatosensory cortex [42]. In agreement with previous studies, our MR analysis corroborates the anxiolytic properties of bumetanide, offering further validation and highlighting the utility of our method in drug repurposing.

Prednisolone, a glucocorticoid with an extensive medical history, is primarily utilized to combat inflammation and autoimmune diseases. However, it carries a spectrum of side effects, necessitating a careful assessment of potential risks and benefits under the guidance of a physician. MR analysis suggests that prednisolone may mitigate anxiety disorders. In a study focusing on post-COVID-19 symptoms, the combination of prednisolone with B vitamins was found to alleviate anxiety and fatigue in patients [43]. Another possibility is that inflammation and autoimmune diseases may trigger anxiety symptoms, and since prednisolone can control inflammation, it might theoretically help alleviate these symptoms. However, considering the potential side effects associated with corticosteroids, the use of prednisolone to treat anxiety may not be ideal [44]. An exception is the treatment of autoimmune diseases that may co-occur with mental disorders, such as anxiety, where the use of prednisolone to address the primary condition and indirectly alleviate anxiety, as in the case of neuropsychiatric systemic lupus erythematosus, is advantageous [45].

Bumetanide and prednisolone may serve as adjunctive therapies for specific subgroups, such as treatment-resistant patients or those with comorbidities. However, further clinical studies are required to establish optimal dosing regimens and treatment durations to ensure safety and reduce adverse effects.

Thiamine or vitamin B_1_ has been implicated in the mitigation of dementia, particularly in nutritional or chronic alcoholic encephalopathy [46]. So far, it hasn't exhibited comparable impacts in other dementias such as AD. However, MR study of dementia outcomes from the FinnGen database, which mainly includes Alzheimer's, vascular, and Parkinson's dementias, shows no association with nutritional or chronic alcoholic brain disorders. Vitamin B_1_ can counteract oxidative stress, modulate apoptosis and inflammation, sustain energy metabolism, and hinder the accumulation of β-amyloid protein [47-50]. Recent studies suggest that thiamine may exert its protective effects by preventing brain iron overload, activating the Nrf2/ARE pathway, and reversing the hypometabolism of brain glucose [51]. Additionally, thiamine supplementation and its derivatives have been shown to improve dementia symptoms significantly [52]. This contradiction means that despite inconsistent clinical research findings, vitamin B_1_ might still be related to non-nutritional brain diseases.

Creon e/c granules in capsules is a digestive system medication, while epilepsy is a neurological condition, and there is no current report of a causal relationship between them. The main components of Creon e/c granules in the capsule include pancreatic enzymes, such as protease, lipase, and amylase, which are used to treat pancreatic insufficiency for various reasons [53]. It serves as a pancreatic enzyme replacement therapy, primarily aiding digestion and absorption. Although the mechanism by which Creon affects epilepsy is unclear, studies suggest that it may involve the gut-brain axis and metabolic regulation [54, 55]. In epilepsy patients, disrupted gut microbiota allows abnormal metabolites to cross the intestinal barrier into the bloodstream [56]. Creon's pancreatic lipase breaks down dietary fiber to produce short-chain fatty acids (SCFAs), which can inhibit abnormal discharges in the temporal lobe *via* the vagus nerve [57].

Additionally, its pancreatic protease enhances protein digestion and may indirectly reduce the pro-epileptic factor IL-6. The lipase also improves lipid absorption, increasing vitamin D levels (epilepsy control associated) and regulating branched-chain amino acid metabolism [58, 59]. These biological processes provide a theoretical basis for Creon's potential cross-indication effects, but further clinical research is needed to confirm these mechanisms.

Pentasa SR 250 mg m/r tablet, on the other hand, is a slow-release formulation of mesalazine, a 5-aminosalicylic acid drug mainly used for the treatment of non-specific Inflammatory Bowel Disease (IBD). Neurological complications of IBDs are common yet often underdiagnosed, with studies showing a higher incidence of demyelinating diseases such as multiple sclerosis in patients with IBD [60]. Both IBD and MS are autoimmune diseases, possibly sharing a genetic basis related to immunity. Mesalazine might alleviate multiple sclerosis by inhibiting inflammation, reducing T-cell activation, regulating immune responses, affecting gut microbiota, and providing neuroprotection [61-63]. The effect of mesalazine is also based on the theory of the gut-brain axis, wherein it acts indirectly. Its efficacy, however, requires further research to be fully validated.

Zolmitriptan typically treats acute migraine attacks by activating the 5-HT1B/1D receptors, constricting intracranial blood vessels, and inhibiting inflammatory neuropeptides [64]. The MR results suggest that zolmitriptan reduces the risk of stroke, and LDSC indicates a negative genetic correlation between the two. However, no clinical research supports these findings. Conversely, zolmitriptan is contraindicated in patients with uncontrolled blood pressure, and because of the presence of 5-HT1B receptors in the coronary arteries and platelets, zolmitriptan may even cause coronary artery spasm and platelet aggregation, increasing the risk of stroke [65, 66]. Nevertheless, pain, as a stress response, can significantly increase the risk of cardiovascular events, and zolmitriptan's rapid relief of acute migraine attacks could positively impact the overall cardiovascular health of some high-risk patients by effectively controlling migraines [67]. Notably, not a single research has confirmed zolmitriptan worsens stroke outcomes. Dosage and timing may be critical in its impact, and these could be explored in future studies.

In this study, the MR analysis has several limitations. The data derived from a European population suggests the need for further research with diverse racial groups to broaden the applicability of our conclusions. The reliance on verbal interviews for medication usage introduces potential subjectivity in quantification, and the limited number of cases results in a low sample size, which may skew the effect estimates. These issues could be addressed by using clinician-verified clinical records and integrating multiple datasets in future research. Additionally, while we controlled for known confounders, unmeasured factors such as medication adherence and environmental influences were not fully addressed; thus, meta-analyses and covariate adjustments could enhance the accuracy of our results [13, 24]. Most of our results are corroborated by existing literature, supporting the utility of MR analyses for drug repurposing. However, caution is warranted for results lacking supporting evidence or those that contradict biological principles, such as the purported causal link between zolmitriptan and stroke risk, which necessitates clinical and experimental validation. Furthermore, the potential nonlinearity in medication dosage underscores the need for a stratified analysis with additional data to ensure the validity of our conclusions.

Drug repurposing offers cost-saving potential by bypassing early-stage development, though challenges such as regulatory barriers, post-market monitoring costs, and dose optimization must be addressed. Streamlined approval processes and rigorous clinical validation can mitigate delays caused by strict reapproval requirements. Patients, especially in mental health, often favor repurposed drugs due to established safety profiles. This strategy enhances access to affordable treatments in resource-limited settings, advancing health equity. Prioritizing cost-effective repurposing is critical for reducing global disease burdens.

## CONCLUSION

This study utilizes the MR method to conduct an in-depth exploration into the repurposing of currently available medications. A significant portion of our research findings demonstrate alignment with the existing body of literature, thereby emphasizing that MR emerges as a highly promising strategy for unearthing novel therapeutic applications for well-established drugs. Nevertheless, it is important to note that due to the comparatively limited sample size of the medication data, the complexity of potential confounding factors, as well as the possibility of nonlinear drug effects, the obtained results of this study should merely be regarded as initial and provisional indications. To guarantee the precision of these findings and successfully transform this research into concrete therapeutic advantages that can be tangibly realized, further extensive clinical and experimental validations are unquestionably essential. Moreover, to achieve clinical implementation, it is crucial to address regulatory hurdles, cost-effectiveness, and patient acceptance. By addressing these challenges, we can unlock the full potential of MR to contribute significantly to the development of new therapeutic strategies and improve public health outcomes.

## Figures and Tables

**Fig. (1):**
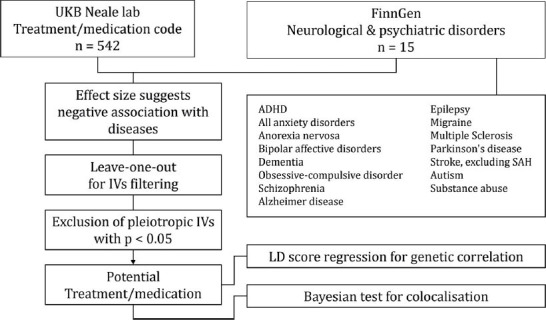
The overall design of Mendelian randomization analysis in the present study.

**Fig. (2):**
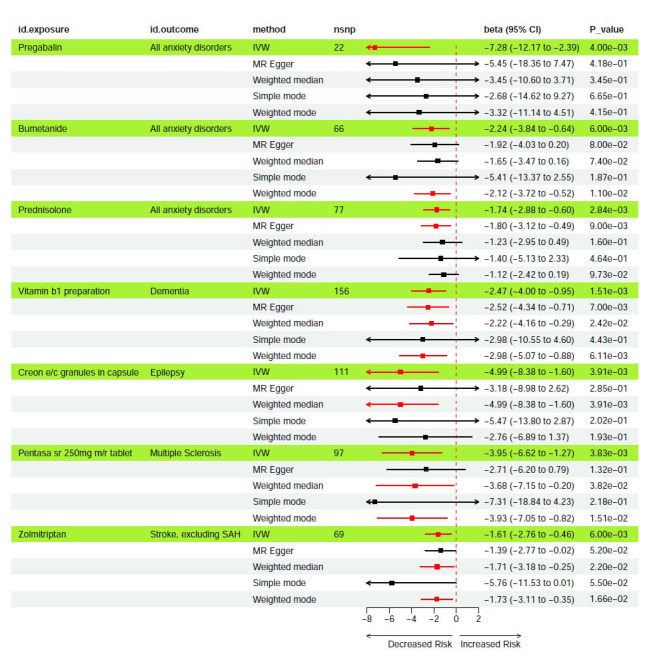
The forest plot illustrates negative causal associations between medications and neuropsychiatric disorders.

**Table 1:** Data sources for treatment/medication as exposure and neuropsychiatric disorders as outcomes.

**Traits**	**Label**	**Consortium**	**Participants**			**Web Source**	**Genome-wide Significance**
			**Ncase**	**Ncontrol**	**Total**		
Treatment/medication code	-	UKBB Neale lab	361141 in total, ncase can be found in the source data			https://www.nealelab.is/uk-biobank	5e-8; 5e-6 when IVs<3
ADHD	Finngen_R10_F5_ADHD	FinnGen	2788	405153	407941	https://www.finngen.fi/en	-
All anxiety disorders	Finngen_R10_F5_ALLANXIOUS		27664	368054	395718		
Anorexia nervosa	Finngen_R10_F5_ANOREX		2115	400510	402625		
Bipolar affective disorders	Finngen_R10_F5_BIPO		7569	359290	366859		
Dementia	Finngen_R10_F5_DEMENTIA		20338	391843	412181		
Obsessive-compulsive disorder	Finngen_R10_F5_OCD		2175	368054	370229		
Schizophrenia	Finngen_R10_F5_SCHZPHR		6708	398386	405094		
Alzheimer disease	Finngen_R10_G6_ALZHEIMER		10520	401661	412181		
Epilepsy	Finngen_R10_G6_EPLEPSY		12891	312803	325694		
Migraine	Finngen_R10_G6_MIGRAINE		5229	367565	372794		
Multiple Sclerosis	Finngen_R10_G6_MS		2409	408561	410970		
Parkinson's disease	Finngen_R10_G6_PARKINSON		4681	407500	412181		
Stroke, excluding SAH	Finngen_R10_I9_STR		27497	371723	399220		
Autism	Finngen_R10_KRA_PSY_ AUTISM_EXMORE		646	301879	302525		
Substance abuse	Finngen_R10_KRA_PSY_ SUBSTANCE_EXMORE		25459	301879	327338		

**Table 2:** Sensitivity tests for the mendelian randomization analyses.

**Id.exposure**	**Id.outcome**	**Method**	**Heterogeneity Test**				**Pleiotropy Test**		
			**Cochran's Q Test**			**MR_PRESSO Global Test**			
			**Q**	**Q_df**	**Q_pval**	**pval**	**Egger_ Intercept**	**se**	**pval**
Pregabalin	All anxiety disorders	MR Egger	15.229	20	0.763	0.782	-0.014	0.046	0.767
		IVW	15.319	21	0.807				
Bumetanide	All anxiety disorders	MR Egger	77.865	64	0.114	0.147	-0.005	0.012	0.650
		IVW	78.117	65	0.127				
Prednisolone	All anxiety disorders	MR Egger	73.684	75	0.521	0.567	0.001	0.005	0.845
		IVW	73.722	76	0.553				
Vitamin B_1_ preparation	Dementia	MR Egger	158.703	154	0.381	0.456	0.001	0.009	0.924
		IVW	158.712	155	0.402				
Creon e/c granules in capsule	Eplepsy	MR Egger	90.964	109	0.894	0.902	-0.011	0.015	0.452
		IVW	91.533	110	0.899				
Pentasa sr 250 mg m/r tablet	Multiple Sclerosis	MR Egger	94.096	95	0.507	0.564	-0.030	0.027	0.281
		IVW	95.272	96	0.502				
Zolmitriptan	Stroke, excluding SAH	MR Egger	52.242	67	0.907	0.936	-0.006	0.011	0.572
		IVW	52.564	68	0.916				

## Data Availability

All genomic and clinical data utilized in this paper, specifically the GWAS data, can be retrieved from public repositories in the UK Biobank Neale Lab database and the FinnGen database.

## LIST OF ABBREVIATIONS

AD: Alzheimer's Disease
ADHD: Attention Deficit Hyperactivity Disorder
ALS: Amyotrophic Lateral Sclerosis
COLOC: Bayesian Colocalization
GWAS: Genome-wide Association Study
IBD: Inflammatory Bowel Disease
IVW: Inverse Variance Weighted
IVs: Instrumental Variants
LDS: Linkage Disequilibrium Score Correlation
MR: Mendelian Randomization
MS: Multiple Sclerosis
PD: Parkinson's Disease
PPH4: Posterior Probability of H4
Rg: Genetic Correlation Coefficient
SAH: Subarachnoid Hemorrhage
