# Supplementary material for: Mendelian Randomization Uncovers Potential Repurposable Medications for Neuropsychiatric Disorders
Source: Curr Neuropharmacol. 2025 Jun 10;24(2):241–53. doi: 10.2174/011570159X368382250527073353 (PMC13054731; doi:10.2174/011570159X368382250527073353)
Supplement: Supplementary file 1 [file CN-24-2-241_SD1.pdf]

Supplementary Material

Mendelian Randomization Uncovers Potential Repurposable Medications for Neuropsychiatric Disorders

Xiao Xiao[1,2], Tingyu Li[3], Qiang Wang[3], Linbo Gao[3,*], Shanling Liu[1,2,*] and Lin Zhang[3,*]

[1]Department of Medical Genetics, West China Second University Hospital, Sichuan University, Chengdu, Sichuan, China; [2]Key Laboratory of Birth Defects and Related Diseases of Women and Children (Sichuan University), Ministry of Education, Chengdu, Sichuan, China; [3]Laboratory of Molecular Translational Medicine, Center for Translational Medicine, Key Laboratory of Birth Defects and Related Diseases of Women and Children (Sichuan University), Ministry of Education, West China Second University Hospital, Sichuan University, Chengdu, People's Republic of China

1875-6190/26 $65.00+.00 **© 2026 Bentham Science Publishers**

a.

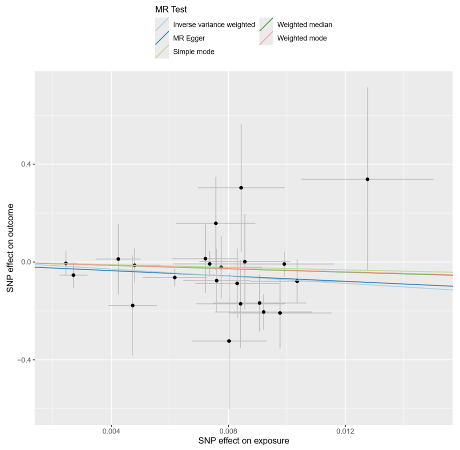
MR Test
Inverse variance weighted
MR Egger
Simple mode
Weighted median
Weighted mode
0.4
0.0
−0.4
SNP effect on outcome
0.004
0.008
0.012
SNP effect on exposure

b.

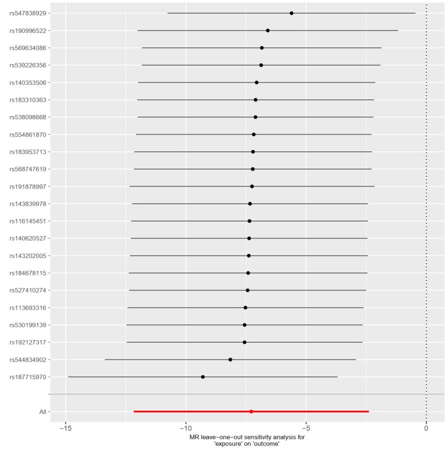
rs547838929
rs190996522
rs569634086
rs539226356
rs140353506
rs183310363
rs538098668
rs554861870
rs183953713
rs568747619
rs191878997
rs143839978
rs116145451
rs140620527
rs143202005
rs184678115
rs527410274
rs113693316
rs530199139
rs192127317
rs544834902
rs187715970
All
−15
−10
−5
0
MR leave−one−out sensitivity analysis for
'exposure' on 'outcome'

c.

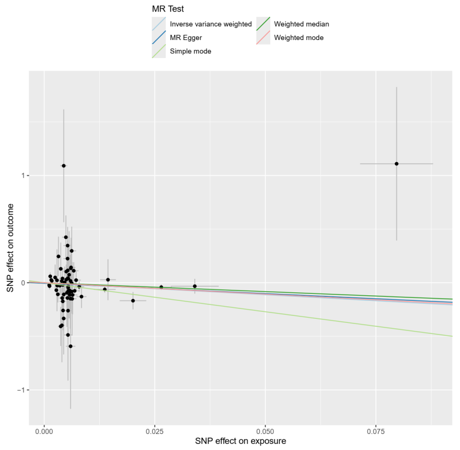
MR Test
Inverse variance weighted
MR Egger
Simple mode
Weighted median
Weighted mode
1
0
−1
SNP effect on outcome
0.000
0.025
0.050
0.075
SNP effect on exposure

d.

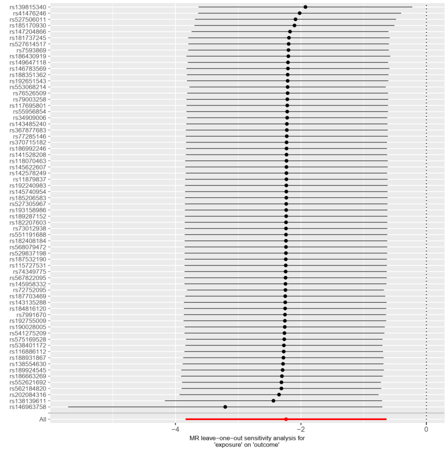
All
−4
−2
0
MR leave−one−out sensitivity analysis for
'exposure' on 'outcome'

e.

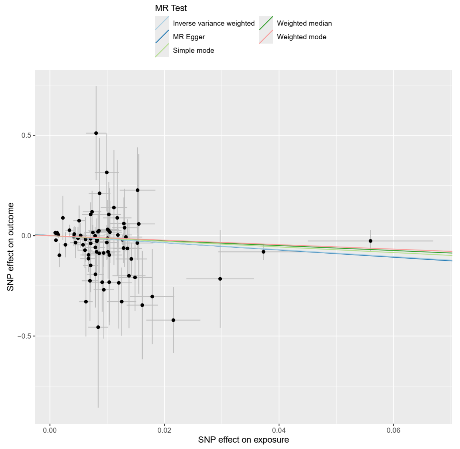
MR Test
Inverse variance weighted
MR Egger
Simple mode
Weighted median
Weighted mode
0.5
0.0
−0.5
SNP effect on outcome
0.00
0.02
0.04
0.06
SNP effect on exposure

f.

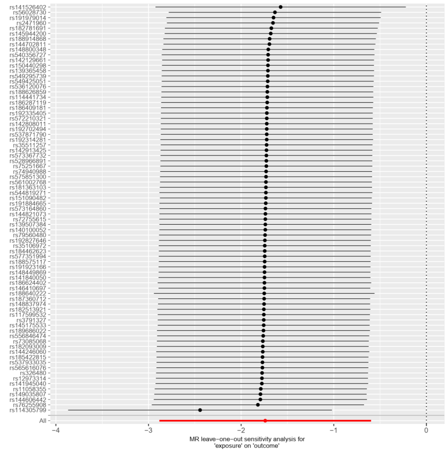
All
−4
−3
−2
−1
0
MR leave−one−out sensitivity analysis for
'exposure' on 'outcome'

g.

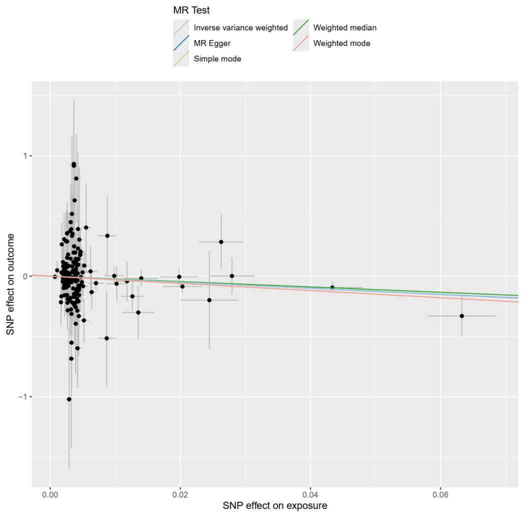
MR Test
Inverse variance weighted
MR Egger
Simple mode
Weighted median
Weighted mode
SNP effect on outcome
SNP effect on exposure

h.

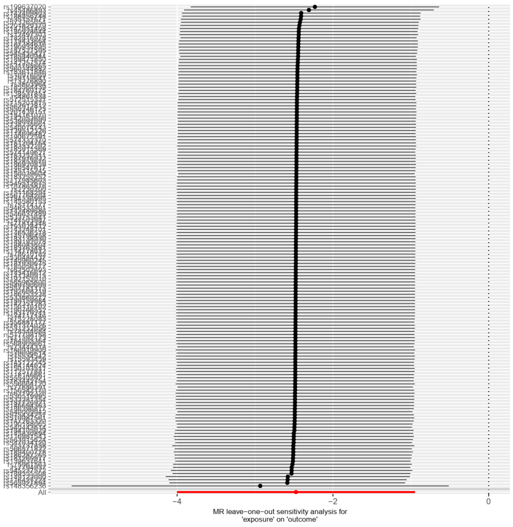
All
MR leave–one–out sensitivity analysis for
'exposure' on 'outcome'

i.

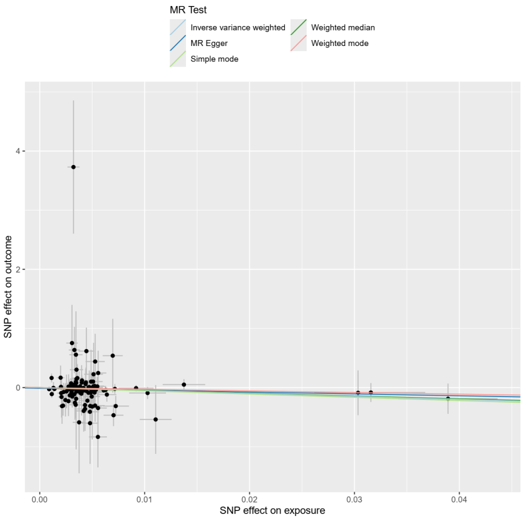
MR Test
Inverse variance weighted
MR Egger
Simple mode
Weighted median
Weighted mode
SNP effect on outcome
SNP effect on exposure

j.

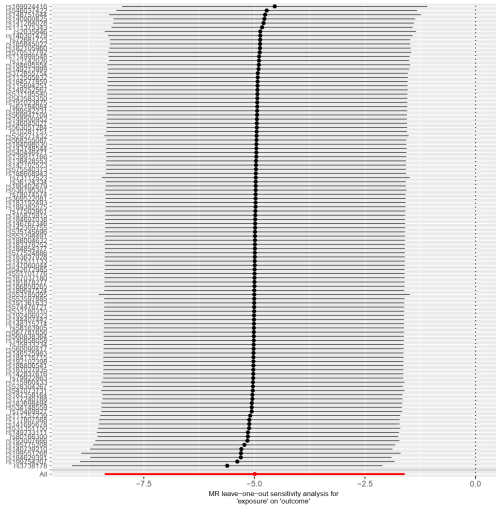
All
MR leave–one–out sensitivity analysis for
'exposure' on 'outcome'

k.

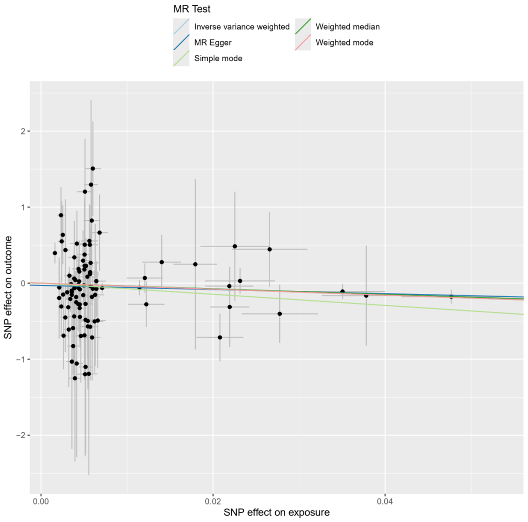
MR Test
Inverse variance weighted
MR Egger
Simple mode
Weighted median
Weighted mode
SNP effect on outcome
SNP effect on exposure

l.

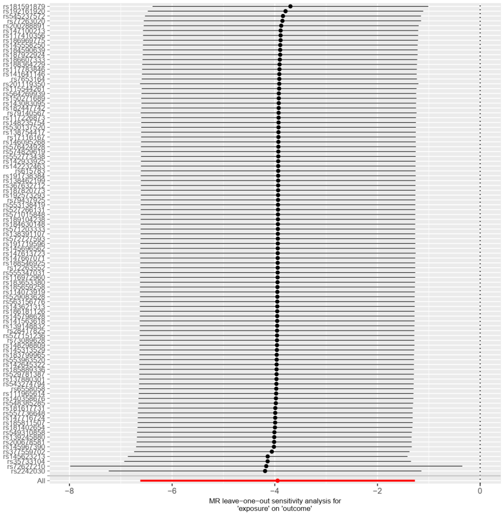
All
MR leave–one–out sensitivity analysis for
'exposure' on 'outcome'

m.

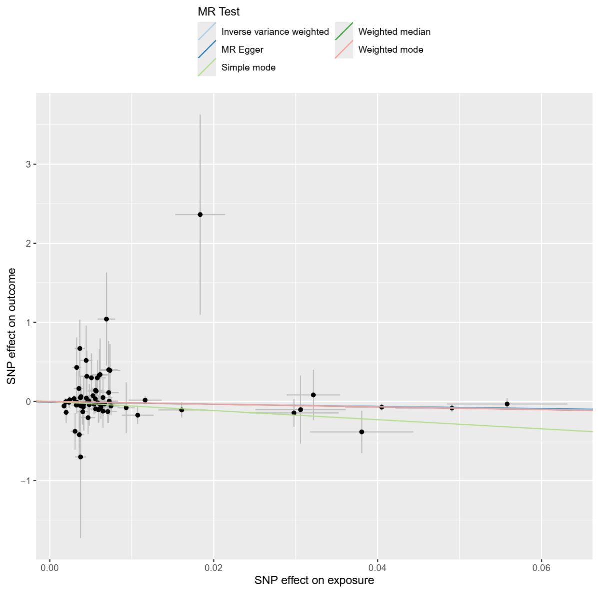

n.

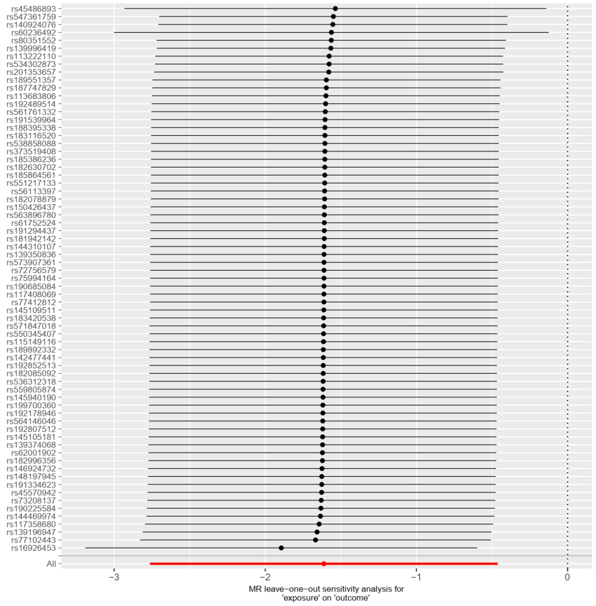

Figure S1. Scatterplot (left) and Leave-One-Out Analysis (right) for MR Positive Results. Figures a-n respectively show the scatterplot and leave-one-out analysis of causal relationships in Mendelian randomization (MR) analysis for the following exposure-outcome pairs: pregabalin on all anxiety disorders (a-b); bumetanide on all anxiety disorders (c-d); prednisolone on all anxiety disorders (e-f); vitamin b1 preparation on dementia (g-h); creon e/c granules in capsule on epilepsy (i-j); pentasa sr 250mg m/r tablet on multiple sclerosis (k-l); and zolmitriptan on stroke, excluding SAH (m-n).

a.

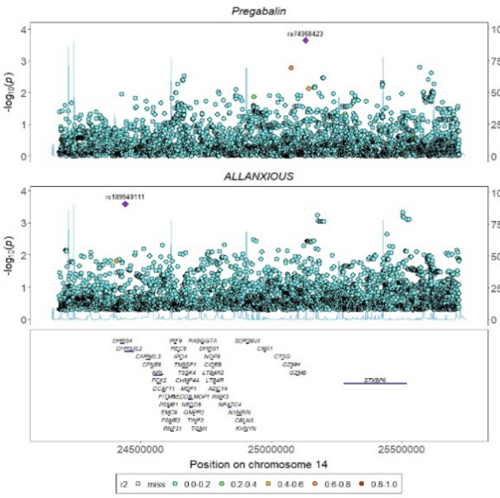
Pregabalin
rs74968423
ALLANXIOUS
rs189949111
-log10(p)
Recomb. rate
Position on chromosome 14
r2 miss 0.0-0.2 0.2-0.4 0.4-0.6 0.6-0.8 0.8-1.0

b.

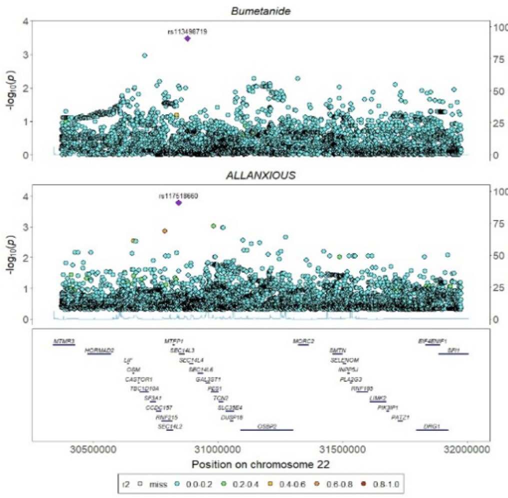
Bumetanide
rs113496719
ALLANXIOUS
rs117518660
-log10(p)
Recomb. rate
Position on chromosome 22
r2 miss 0.0-0.2 0.2-0.4 0.4-0.6 0.6-0.8 0.8-1.0

c.

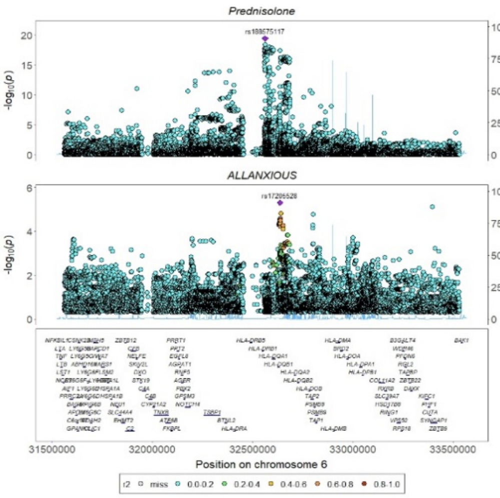
Prednisolone
rs188675117
ALLANXIOUS
rs17206528
-log10(p)
Recomb. rate
Position on chromosome 6
r2 miss 0.0-0.2 0.2-0.4 0.4-0.6 0.6-0.8 0.8-1.0

d.

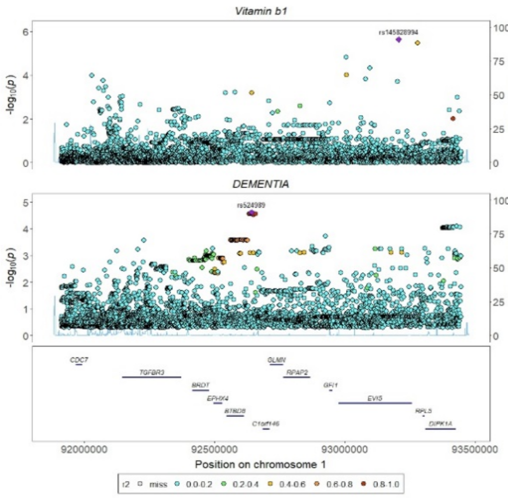
Vitamin b1
rs145828994
DEMENTIA
rs524989
-log10(p)
Recomb. rate
Position on chromosome 1
r2 miss 0.0-0.2 0.2-0.4 0.4-0.6 0.6-0.8 0.8-1.0

e.

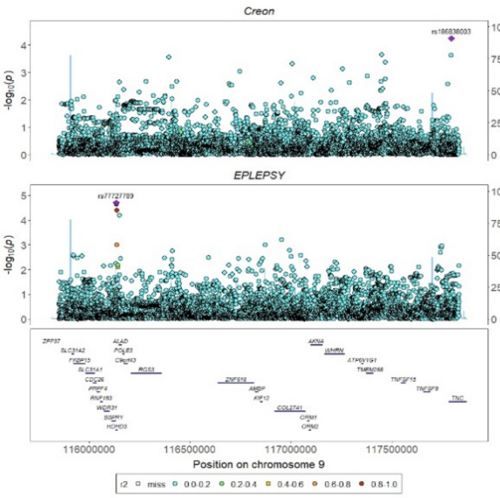
Creon
rs186836093
EPLEPSY
rs77727789
-log10(p)
Recomb. rate
Position on chromosome 9
r2 miss 0.0-0.2 0.2-0.4 0.4-0.6 0.6-0.8 0.8-1.0

f.

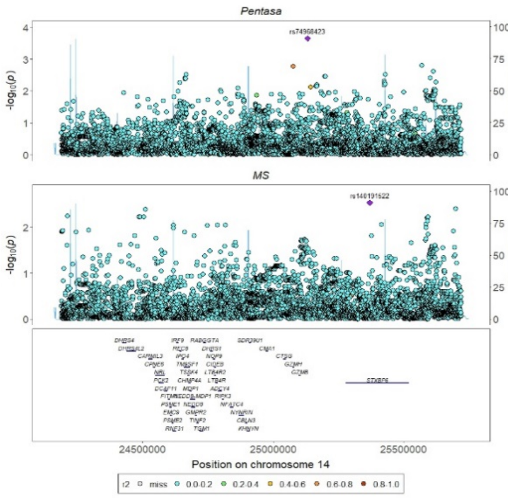
Pentasa
rs74968423
MS
rs140191522
-log10(p)
Recomb. rate
Position on chromosome 14
r2 miss 0.0-0.2 0.2-0.4 0.4-0.6 0.6-0.8 0.8-1.0

g.

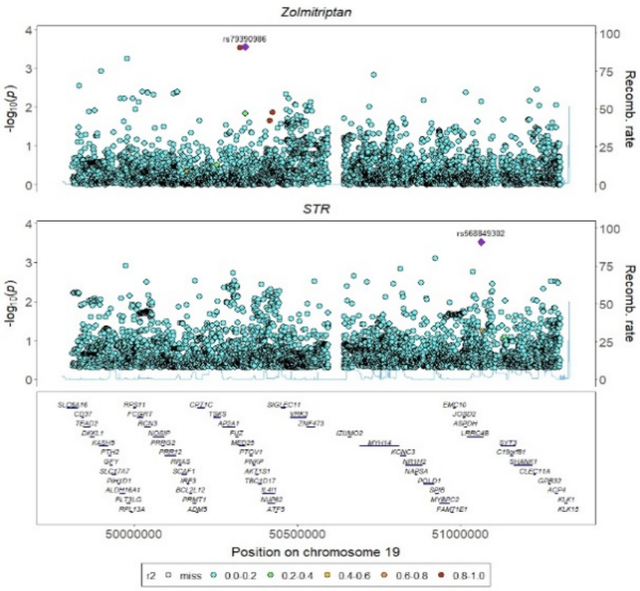

Fig. (S2). Bayesian colocalization analysis plot showing the interaction between treatment/medication response and neuropsychiatric disorders within a specific genomic region. (a) Pregabalin vs All anxiety disorders; (b) Bumetanide vs. all anxiety disorders; (c) Prednisolone vs. all anxiety disorders; (d) Vitamin B1 preparation vs. dementia; (e) Creon e/c granules in capsule vs Epilepsy; (f) Pentasa SR 250mg tablet vs. multiple sclerosis; (g) Zolmitriptan vs. stroke (excluding SAH).

Table S1. Basic information of exposures and its genome-wide significance.

Phenotype Code	Phenotype Description	Genome-wide Significance
20003_1140851088	Treatment/medication code: senokot 7.5mg tablet	5.00E-08
20003_1140851812	Treatment/medication code: gtn 400micrograms spray	5.00E-08
20003_1140852756	Treatment/medication code: vitamin a	5.00E-08
20003_1140852948	Treatment/medication code: calcium+vitamin d 500units tablet	5.00E-06
20003_1140852976	Treatment/medication code: multivitamins	5.00E-06
20003_1140856342	Treatment/medication code: syndol tablet	5.00E-08
20003_1140858452	Treatment/medication code: hepacon b12 1mg/1ml injection	5.00E-08
20003_1140860696	Treatment/medication code: lisinopril	5.00E-06
20003_1140860728	Treatment/medication code: quinapril	5.00E-08
20003_1140860750	Treatment/medication code: captopril	5.00E-08
20003_1140860802	Treatment/medication code: coversyl 2mg tablet	5.00E-08
20003_1140860806	Treatment/medication code: ramipril	5.00E-06
20003_1140860834	Treatment/medication code: glyceryl trinitrate	5.00E-08
20003_1140860840	Treatment/medication code: nitrolingual 400micrograms spray	5.00E-08
20003_1140860904	Treatment/medication code: trandolapril	5.00E-08
20003_1140860954	Treatment/medication code: isosorbide mononitrate	5.00E-08
20003_1140860976	Treatment/medication code: imdur 60mg durule	5.00E-08
20003_1140861088	Treatment/medication code: nifedipine	5.00E-08
20003_1140861090	Treatment/medication code: adalat 5mg capsule	5.00E-08
20003_1140861120	Treatment/medication code: coracten sr 10mg m/r capsule	5.00E-08
20003_1140861128	Treatment/medication code: tildiem 60mg m/r tablet	5.00E-08
20003_1140861138	Treatment/medication code: adizem-60 m/r tablet	5.00E-08
20003_1140861166	Treatment/medication code: dilzem sr 60mg long acting m/r capsule	5.00E-08
20003_1140861202	Treatment/medication code: istin 5mg tablet	5.00E-08
20003_1140861276	Treatment/medication code: lacidipine	5.00E-08
20003_1140861778	Treatment/medication code: dipyridamole	5.00E-08
20003_1140861780	Treatment/medication code: persantin 25mg tablet	5.00E-08
20003_1140861806	Treatment/medication code: aspirin 75mg tablet	5.00E-06
20003_1140861924	Treatment/medication code: bezafibrate	5.00E-08
20003_1140861936	Treatment/medication code: questran 4g/sachet powder	5.00E-08
20003_1140861954	Treatment/medication code: fenofibrate	5.00E-08
20003_1140861958	Treatment/medication code: simvastatin	5.00E-06
20003_1140861998	Treatment/medication code: ventolin 100micrograms inhaler	5.00E-06
20003_1140862060	Treatment/medication code: ventolin 2mg tablet	5.00E-08
20003_1140862086	Treatment/medication code: salamol 100micrograms inhaler	5.00E-08
20003_1140862144	Treatment/medication code: salmeterol	5.00E-08
20003_1140862148	Treatment/medication code: serevent 25mcg inhaler	5.00E-08
20003_1140862168	Treatment/medication code: bricanyl 250mcg inhaler	5.00E-08
20003_1140862236	Treatment/medication code: atrovent 20micrograms inhaler	5.00E-08

Phenotype Code	Phenotype Description	Genome-wide Significance
20003_1140862266	Treatment/medication code: phyllocontin continus 225mg m/r tablet	5.00E-08
20003_1140862380	Treatment/medication code: becloforte 250micrograms inhaler	5.00E-08
20003_1140862382	Treatment/medication code: becotide 50 inhaler	5.00E-06
20003_1140862438	Treatment/medication code: uniphyllin continus 200mg m/r tablet	5.00E-08
20003_1140862476	Treatment/medication code: beclazone 50 inhaler	5.00E-08
20003_1140862526	Treatment/medication code: sodium cromoglycate	5.00E-08
20003_1140862572	Treatment/medication code: budesonide	5.00E-08
20003_1140862574	Treatment/medication code: pulmicort ls 50micrograms inhaler	5.00E-08
20003_1140862628	Treatment/medication code: piriton 4mg tablet	5.00E-08
20003_1140862770	Treatment/medication code: zirtek 10mg tablet	5.00E-08
20003_1140862772	Treatment/medication code: loratadine	5.00E-06
20003_1140862776	Treatment/medication code: clarityn 10mg tablet	5.00E-08
20003_1140862944	Treatment/medication code: carbocisteine	5.00E-08
20003_1140862952	Treatment/medication code: mucodyne 375mg capsule	5.00E-08
20003_1140863144	Treatment/medication code: zopiclone	5.00E-08
20003_1140863152	Treatment/medication code: diazepam	5.00E-08
20003_1140863182	Treatment/medication code: nitrazepam	5.00E-08
20003_1140863202	Treatment/medication code: temazepam	5.00E-08
20003_1140863302	Treatment/medication code: lorazepam	5.00E-08
20003_1140864070	Treatment/medication code: kapake tablet	5.00E-08
20003_1140864184	Treatment/medication code: dovonex 50micrograms/g cream	5.00E-08
20003_1140864286	Treatment/medication code: flixotide 25micrograms inhaler	5.00E-08
20003_1140864734	Treatment/medication code: combivent inhaler	5.00E-08
20003_1140864752	Treatment/medication code: lansoprazole	5.00E-06
20003_1140864950	Treatment/medication code: bisoprolol fumarate+hydrochlorothiazide 10mg/6.25mg tablet	5.00E-08
20003_1140864952	Treatment/medication code: lisinopril+hydrochlorothiazide 10mg/12.5mg tablet	5.00E-08
20003_1140864992	Treatment/medication code: tramadol	5.00E-06
20003_1140865000	Treatment/medication code: zydol 50mg capsule	5.00E-08
20003_1140865010	Treatment/medication code: viscotears liquid eye gel	5.00E-08
20003_1140865016	Treatment/medication code: zolpidem	5.00E-08
20003_1140865336	Treatment/medication code: spasmonal 60mg capsule	5.00E-08
20003_1140865350	Treatment/medication code: fybogel mebeverine sachet	5.00E-08
20003_1140865354	Treatment/medication code: gaviscon liquid	5.00E-06
20003_1140865382	Treatment/medication code: merbentyl 10mg tablet	5.00E-08
20003_1140865394	Treatment/medication code: hyoscine butylbromide	5.00E-08
20003_1140865396	Treatment/medication code: buscopan 10mg tablet	5.00E-08
20003_1140865414	Treatment/medication code: peppermint oil product	5.00E-08
20003_1140865416	Treatment/medication code: colpermin 0.2ml m/r gel e/c capsule	5.00E-08
20003_1140865426	Treatment/medication code: cimetidine	5.00E-08
20003_1140865564	Treatment/medication code: imodium 2mg capsule	5.00E-08

Phenotype Code	Phenotype Description	Genome-wide Significance
20003_1140865578	Treatment/medication code: mesalazine	5.00E-08
20003_1140865580	Treatment/medication code: asacol 400mg e/c tablet	5.00E-08
20003_1140865588	Treatment/medication code: pentasa sr 250mg m/r tablet	5.00E-08
20003_1140865634	Treatment/medication code: omeprazole	5.00E-06
20003_1140865654	Treatment/medication code: codeine phosphate+kaolin 10mg/3g/10ml mixture	5.00E-08
20003_1140865668	Treatment/medication code: sulphasalazine	5.00E-08
20003_1140865670	Treatment/medication code: salazopyrin 500mg tablet	5.00E-08
20003_1140865716	Treatment/medication code: senna	5.00E-08
20003_1140865752	Treatment/medication code: ispaghula husk	5.00E-08
20003_1140865786	Treatment/medication code: bisacodyl	5.00E-08
20003_1140865800	Treatment/medication code: lactulose product	5.00E-08
20003_1140865872	Treatment/medication code: magnesium citrate	5.00E-08
20003_1140866026	Treatment/medication code: creon e/c granules in capsule	5.00E-08
20003_1140866078	Treatment/medication code: indapamide	5.00E-08
20003_1140866116	Treatment/medication code: frusemide	5.00E-08
20003_1140866122	Treatment/medication code: bendrofluazide	5.00E-06
20003_1140866236	Treatment/medication code: spironolactone	5.00E-08
20003_1140866280	Treatment/medication code: bumetanide	5.00E-08
20003_1140866466	Treatment/medication code: securon 40mg tablet	5.00E-08
20003_1140866738	Treatment/medication code: atenolol	5.00E-06
20003_1140866800	Treatment/medication code: half-inderal la 80mg m/r capsule	5.00E-08
20003_1140866804	Treatment/medication code: inderal 10mg tablet	5.00E-08
20003_1140867444	Treatment/medication code: risperidone	5.00E-08
20003_1140867490	Treatment/medication code: lithium product	5.00E-08
20003_1140867504	Treatment/medication code: priadel 200mg m/r tablet	5.00E-08
20003_1140867726	Treatment/medication code: lofepramine	5.00E-08
20003_1140867818	Treatment/medication code: nortriptyline	5.00E-08
20003_1140867876	Treatment/medication code: prozac 20mg capsule	5.00E-08
20003_1140867878	Treatment/medication code: sertraline	5.00E-06
20003_1140867888	Treatment/medication code: paroxetine	5.00E-08
20003_1140867998	Treatment/medication code: motilium 10mg tablet	5.00E-08
20003_1140868064	Treatment/medication code: serc-8 tablet	5.00E-08
20003_1140868080	Treatment/medication code: cyclizine	5.00E-08
20003_1140868170	Treatment/medication code: prochlorperazine	5.00E-08
20003_1140868172	Treatment/medication code: stemetil 5mg tablet	5.00E-08
20003_1140868226	Treatment/medication code: aspirin	5.00E-06
20003_1140868364	Treatment/medication code: prednisone	5.00E-08
20003_1140868426	Treatment/medication code: triamcinolone	5.00E-08
20003_1140868772	Treatment/medication code: didronel 200mg tablet	5.00E-08
20003_1140869848	Treatment/medication code: methotrexate	5.00E-06
20003_1140869930	Treatment/medication code: azathioprine	5.00E-08

Phenotype Code	Phenotype Description	Genome-wide Significance
20003_1140870306	Treatment/medication code: iron+folic acid	5.00E-08
20003_1140870390	Treatment/medication code: ferrous sulphate	5.00E-06
20003_1140870422	Treatment/medication code: folic acid product	5.00E-06
20003_1140870488	Treatment/medication code: forceval capsule	5.00E-08
20003_1140870570	Treatment/medication code: vitamin b12 preparation	5.00E-08
20003_1140870788	Treatment/medication code: calcium salts	5.00E-06
20003_1140870856	Treatment/medication code: calcichew 1.25g chewable tablet	5.00E-08
20003_1140870862	Treatment/medication code: calcichew forte 2.5g chewable tablet	5.00E-08
20003_1140870954	Treatment/medication code: vitamin d product	5.00E-08
20003_1140870956	Treatment/medication code: alfacalcidol	5.00E-08
20003_1140871024	Treatment/medication code: vitamin b compound tablet	5.00E-06
20003_1140871050	Treatment/medication code: calcichew d3 tablet	5.00E-06
20003_1140871052	Treatment/medication code: calcium carbonate+cholecalciferol 1.25g/5micrograms tablet	5.00E-08
20003_1140871112	Treatment/medication code: vitamin e product	5.00E-06
20003_1140871168	Treatment/medication code: voltarol 25mg e/c tablet	5.00E-08
20003_1140871188	Treatment/medication code: etodolac	5.00E-08
20003_1140871196	Treatment/medication code: lodine 200mg tablet	5.00E-08
20003_1140871266	Treatment/medication code: arthrotec tablet	5.00E-08
20003_1140871310	Treatment/medication code: ibuprofen	5.00E-06
20003_1140871336	Treatment/medication code: indomethacin	5.00E-08
20003_1140871462	Treatment/medication code: naproxen	5.00E-06
20003_1140871472	Treatment/medication code: naprosyn 250mg tablet	5.00E-08
20003_1140871506	Treatment/medication code: ketoprofen	5.00E-08
20003_1140871542	Treatment/medication code: mefenamic acid	5.00E-08
20003_1140871666	Treatment/medication code: piroxicam	5.00E-08
20003_1140871680	Treatment/medication code: tylex capsule	5.00E-08
20003_1140871688	Treatment/medication code: solpadol caplet	5.00E-08
20003_1140871692	Treatment/medication code: morphine	5.00E-08
20003_1140871732	Treatment/medication code: buprenorphine	5.00E-08
20003_1140872036	Treatment/medication code: paramax tablet	5.00E-08
20003_1140872072	Treatment/medication code: tegretol 100mg tablet	5.00E-08
20003_1140872112	Treatment/medication code: epanutin 25mg capsule	5.00E-08
20003_1140872150	Treatment/medication code: clonazepam	5.00E-08
20003_1140872198	Treatment/medication code: sodium valproate	5.00E-08
20003_1140872200	Treatment/medication code: epilim 100mg crushable tablet	5.00E-08
20003_1140872228	Treatment/medication code: gabapentin	5.00E-06
20003_1140872290	Treatment/medication code: lamotrigine	5.00E-08
20003_1140872338	Treatment/medication code: madopar 62.5 capsule	5.00E-08
20003_1140872492	Treatment/medication code: nicotine product	5.00E-08
20003_1140872694	Treatment/medication code: flucloxacillin	5.00E-08

Phenotype Code	Phenotype Description	Genome-wide Significance
20003_1140873394	Treatment/medication code: doxycycline	5.00E-08
20003_1140873450	Treatment/medication code: tetracycline	5.00E-08
20003_1140873474	Treatment/medication code: lymecycline	5.00E-08
20003_1140873476	Treatment/medication code: tetralysal 300 capsule	5.00E-08
20003_1140873480	Treatment/medication code: minocycline	5.00E-08
20003_1140873548	Treatment/medication code: oxytetracycline	5.00E-08
20003_1140873608	Treatment/medication code: erythromycin	5.00E-08
20003_1140873966	Treatment/medication code: trimethoprim	5.00E-08
20003_1140874014	Treatment/medication code: metronidazole	5.00E-08
20003_1140874126	Treatment/medication code: nitrofurantoin	5.00E-08
20003_1140874138	Treatment/medication code: ciprofloxacin	5.00E-08
20003_1140874370	Treatment/medication code: aciclovir	5.00E-08
20003_1140874420	Treatment/medication code: quinine	5.00E-06
20003_1140874686	Treatment/medication code: glucophage 500mg tablet	5.00E-08
20003_1140874744	Treatment/medication code: gliclazide	5.00E-06
20003_1140874790	Treatment/medication code: betamethasone	5.00E-08
20003_1140874852	Treatment/medication code: thyroxine sodium	5.00E-08
20003_1140874866	Treatment/medication code: carbimazole	5.00E-08
20003_1140874896	Treatment/medication code: hydrocortisone	5.00E-08
20003_1140874930	Treatment/medication code: prednisolone	5.00E-06
20003_1140875336	Treatment/medication code: nabumetone	5.00E-08
20003_1140875392	Treatment/medication code: plaquenil 200mg tablet	5.00E-08
20003_1140875408	Treatment/medication code: allopurinol	5.00E-06
20003_1140875420	Treatment/medication code: baclofen	5.00E-08
20003_1140875486	Treatment/medication code: colchicine	5.00E-08
20003_1140875632	Treatment/medication code: movelat gel	5.00E-08
20003_1140875840	Treatment/medication code: timolol 0.25% eye drops	5.00E-08
20003_1140875990	Treatment/medication code: hypromellose	5.00E-08
20003_1140876076	Treatment/medication code: flixonase 50micrograms aqueous nasal spray	5.00E-06
20003_1140876136	Treatment/medication code: beconase 50micrograms nasal spray	5.00E-06
20003_1140876146	Treatment/medication code: rhinocort 50micrograms nasal spray	5.00E-08
20003_1140876404	Treatment/medication code: aqueous cream bp	5.00E-08
20003_1140876592	Treatment/medication code: multivitamin+mineral preparations	5.00E-06
20003_1140877630	Treatment/medication code: calcium+ergocalciferol tablet	5.00E-08
20003_1140877826	Treatment/medication code: sodium bicarbonate	5.00E-08
20003_1140877892	Treatment/medication code: voltarol emulgel	5.00E-08
20003_1140878036	Treatment/medication code: diclofenac sodium+misoprostol	5.00E-08
20003_1140878172	Treatment/medication code: tears naturale eye drops	5.00E-08
20003_1140878186	Treatment/medication code: liquifilm tears 1.4% eye drops	5.00E-08
20003_1140878226	Treatment/medication code: diprobase cream	5.00E-08
20003_1140878304	Treatment/medication code: e45 cream	5.00E-08

Phenotype Code	Phenotype Description	Genome-wide Significance
20003_1140878324	Treatment/medication code: oilatum cream	5.00E-08
20003_1140879392	Treatment/medication code: manevac granules	5.00E-08
20003_1140879404	Treatment/medication code: docusate sodium	5.00E-08
20003_1140879406	Treatment/medication code: ranitidine	5.00E-06
20003_1140879424	Treatment/medication code: alverine	5.00E-08
20003_1140879428	Treatment/medication code: mebeverine	5.00E-06
20003_1140879430	Treatment/medication code: domperidone	5.00E-08
20003_1140879464	Treatment/medication code: loperamide	5.00E-08
20003_1140879494	Treatment/medication code: metoclopramide	5.00E-08
20003_1140879540	Treatment/medication code: fluoxetine	5.00E-06
20003_1140879616	Treatment/medication code: amitriptyline	5.00E-06
20003_1140879620	Treatment/medication code: clomipramine	5.00E-08
20003_1140879628	Treatment/medication code: dothiepin	5.00E-08
20003_1140879630	Treatment/medication code: imipramine	5.00E-08
20003_1140879634	Treatment/medication code: trazodone	5.00E-08
20003_1140879644	Treatment/medication code: amantadine	5.00E-08
20003_1140879658	Treatment/medication code: chlorpromazine	5.00E-08
20003_1140879760	Treatment/medication code: bisoprolol	5.00E-06
20003_1140879762	Treatment/medication code: celiprolol	5.00E-08
20003_1140879778	Treatment/medication code: doxazosin	5.00E-06
20003_1140879792	Treatment/medication code: terbutaline	5.00E-08
20003_1140879798	Treatment/medication code: terazosin	5.00E-08
20003_1140879802	Treatment/medication code: amlodipine	5.00E-06
20003_1140879806	Treatment/medication code: diltiazem	5.00E-08
20003_1140879818	Treatment/medication code: metoprolol	5.00E-08
20003_1140879842	Treatment/medication code: propranolol	5.00E-06
20003_1140879854	Treatment/medication code: sotalol	5.00E-08
20003_1140879866	Treatment/medication code: timolol	5.00E-08
20003_1140880072	Treatment/medication code: gamolenic acid	5.00E-08
20003_1140880086	Treatment/medication code: calcipotriol	5.00E-08
20003_1140880956	Treatment/medication code: fentanyl	5.00E-08
20003_1140881320	Treatment/medication code: magnesium carbonate	5.00E-08
20003_1140881446	Treatment/medication code: proctosedyl ointment	5.00E-08
20003_1140881472	Treatment/medication code: lacri-lube eye ointment	5.00E-08
20003_1140881474	Treatment/medication code: normacol granules	5.00E-08
20003_1140881702	Treatment/medication code: adalate 10mg capsule	5.00E-08
20003_1140881856	Treatment/medication code: salbutamol	5.00E-06
20003_1140881882	Treatment/medication code: timoptol 0.25% eye drops	5.00E-08
20003_1140882236	Treatment/medication code: seroxat 20mg tablet	5.00E-08
20003_1140882272	Treatment/medication code: oramorph 10mg/5ml oral solution	5.00E-08
20003_1140882394	Treatment/medication code: paracetamol + codeine	5.00E-08

Phenotype Code	Phenotype Description	Genome-wide Significance
20003_1140882498	Treatment/medication code: penicillin	5.00E-08
20003_1140882618	Treatment/medication code: diprosalic ointment	5.00E-08
20003_1140882626	Treatment/medication code: betnesol 0.1% eye/ear/nose drops	5.00E-08
20003_1140882694	Treatment/medication code: betnovate cream	5.00E-08
20003_1140882728	Treatment/medication code: otomize ear spray	5.00E-08
20003_1140882776	Treatment/medication code: fucibet cream	5.00E-08
20003_1140882782	Treatment/medication code: dermovate cream	5.00E-08
20003_1140882800	Treatment/medication code: eumovate cream	5.00E-08
20003_1140882910	Treatment/medication code: daktacort cream	5.00E-08
20003_1140883066	Treatment/medication code: insulin product	5.00E-06
20003_1140883468	Treatment/medication code: clonidine	5.00E-08
20003_1140883476	Treatment/medication code: procyclidine	5.00E-08
20003_1140883504	Treatment/medication code: cetirizine	5.00E-06
20003_1140883524	Treatment/medication code: cinnarizine	5.00E-08
20003_1140883548	Treatment/medication code: ipratropium	5.00E-08
20003_1140883568	Treatment/medication code: oxybutynin	5.00E-08
20003_1140883656	Treatment/medication code: hydroxyzine	5.00E-08
20003_1140883664	Treatment/medication code: pizotifen	5.00E-08
20003_1140883748	Treatment/medication code: selenium product	5.00E-08
20003_1140883968	Treatment/medication code: carmellose	5.00E-08
20003_1140884308	Treatment/medication code: hydroxychloroquine	5.00E-08
20003_1140884412	Treatment/medication code: sumatriptan	5.00E-08
20003_1140884444	Treatment/medication code: codeine	5.00E-06
20003_1140884464	Treatment/medication code: dihydrocodeine	5.00E-08
20003_1140884488	Treatment/medication code: diclofenac	5.00E-06
20003_1140884516	Treatment/medication code: thyroxine product	5.00E-06
20003_1140884600	Treatment/medication code: metformin	5.00E-06
20003_1140884654	Treatment/medication code: beclomethasone	5.00E-08
20003_1140884672	Treatment/medication code: fludrocortisone	5.00E-08
20003_1140884696	Treatment/medication code: clobetasone	5.00E-08
20003_1140884700	Treatment/medication code: trimovate ointment	5.00E-08
20003_1140888092	Treatment/medication code: elocon cream	5.00E-08
20003_1140888098	Treatment/medication code: fluticasone	5.00E-06
20003_1140888172	Treatment/medication code: mometasone	5.00E-08
20003_1140888266	Treatment/medication code: warfarin	5.00E-06
20003_1140888362	Treatment/medication code: pyridoxine preparation	5.00E-08
20003_1140888366	Treatment/medication code: thiamine preparation	5.00E-08
20003_1140888386	Treatment/medication code: iron product	5.00E-08
20003_1140888390	Treatment/medication code: ferrous salt product	5.00E-08
20003_1140888502	Treatment/medication code: amiodarone	5.00E-08
20003_1140888510	Treatment/medication code: verapamil	5.00E-08

Phenotype Code	Phenotype Description	Genome-wide Significance
20003_1140888512	Treatment/medication code: amiloride	5.00E-08
20003_1140888538	Treatment/medication code: zinc product	5.00E-06
20003_1140888552	Treatment/medication code: enalapril	5.00E-06
20003_1140888556	Treatment/medication code: fosinopril	5.00E-08
20003_1140888560	Treatment/medication code: perindopril	5.00E-06
20003_1140888570	Treatment/medication code: flecainide	5.00E-08
20003_1140888594	Treatment/medication code: fluvastatin	5.00E-08
20003_1140888646	Treatment/medication code: felodipine	5.00E-06
20003_1140888648	Treatment/medication code: pravastatin	5.00E-06
20003_1140888688	Treatment/medication code: betahistine	5.00E-08
20003_1140888758	Treatment/medication code: penicillin v	5.00E-08
20003_1140888762	Treatment/medication code: ismn - isosorbide mononitrate	5.00E-08
20003_1140909368	Treatment/medication code: carvedilol	5.00E-08
20003_1140909482	Treatment/medication code: opticrom allergy eye drops	5.00E-08
20003_1140909578	Treatment/medication code: losec 10mg capsule	5.00E-08
20003_1140909674	Treatment/medication code: cod liver oil capsule	5.00E-06
20003_1140909702	Treatment/medication code: sulfasalazine	5.00E-08
20003_1140909708	Treatment/medication code: furosemide	5.00E-06
20003_1140909726	Treatment/medication code: vitamin c product	5.00E-06
20003_1140909766	Treatment/medication code: multivitamins capsule	5.00E-08
20003_1140909786	Treatment/medication code: beclometasone	5.00E-06
20003_1140909788	Treatment/medication code: sodium cromoglicate	5.00E-08
20003_1140909790	Treatment/medication code: chlorphenamine	5.00E-08
20003_1140909806	Treatment/medication code: dosulepin	5.00E-08
20003_1140909872	Treatment/medication code: vitamin b1 preparation	5.00E-08
20003_1140909874	Treatment/medication code: vitamin b6 preparation	5.00E-08
20003_1140909936	Treatment/medication code: indometacin	5.00E-08
20003_1140909954	Treatment/medication code: acyclovir	5.00E-08
20003_1140910494	Treatment/medication code: b12 - hydroxocobalamin prep	5.00E-08
20003_1140910498	Treatment/medication code: flu - influenza vaccine	5.00E-08
20003_1140910512	Treatment/medication code: ismo - isosorbide mononitrate	5.00E-08
20003_1140910548	Treatment/medication code: iron sulphate	5.00E-08
20003_1140910640	Treatment/medication code: luteine	5.00E-08
20003_1140910698	Treatment/medication code: oil of peppermint	5.00E-08
20003_1140910766	Treatment/medication code: nicorandil	5.00E-08
20003_1140910814	Treatment/medication code: sodium thyroxine	5.00E-08
20003_1140910832	Treatment/medication code: sodium warfarin	5.00E-08
20003_1140911638	Treatment/medication code: kelp+garlic product	5.00E-08
20003_1140911640	Treatment/medication code: lecithin product	5.00E-08
20003_1140911642	Treatment/medication code: tacrolimus	5.00E-08
20003_1140911658	Treatment/medication code: imigran 50mg tablet	5.00E-08

Phenotype Code	Phenotype Description	Genome-wide Significance
20003_1140911682	Treatment/medication code: selenium ace tablet	5.00E-08
20003_1140911698	Treatment/medication code: slozem 120mg m/r capsule	5.00E-08
20003_1140911730	Treatment/medication code: flax oil tablet	5.00E-08
20003_1140911732	Treatment/medication code: garlic product	5.00E-06
20003_1140911734	Treatment/medication code: ginkgo forte tablet	5.00E-06
20003_1140911736	Treatment/medication code: ginseng product	5.00E-08
20003_1140911754	Treatment/medication code: anadin tablet	5.00E-08
20003_1140913292	Treatment/medication code: synalar 1:10 cream	5.00E-08
20003_1140916282	Treatment/medication code: venlafaxine	5.00E-06
20003_1140916288	Treatment/medication code: efexor 37.5mg tablet	5.00E-08
20003_1140916356	Treatment/medication code: losartan	5.00E-06
20003_1140916682	Treatment/medication code: evening primrose oil	5.00E-06
20003_1140916980	Treatment/medication code: zantac 75 tablet	5.00E-08
20003_1140917034	Treatment/medication code: airomir 100micrograms cfc-free inhaler	5.00E-08
20003_1140917428	Treatment/medication code: angitil sr 90 m/r capsule	5.00E-08
20003_1140921600	Treatment/medication code: citalopram	5.00E-06
20003_1140921828	Treatment/medication code: dicloflex 25mg e/c tablet	5.00E-08
20003_1140922174	Treatment/medication code: alendronate sodium	5.00E-06
20003_1140922714	Treatment/medication code: dorzolamide	5.00E-08
20003_1140923276	Treatment/medication code: co-amilozide	5.00E-08
20003_1140923336	Treatment/medication code: co-tenidone	5.00E-08
20003_1140923346	Treatment/medication code: co-codamol	5.00E-06
20003_1140923348	Treatment/medication code: co-proxamol	5.00E-08
20003_1140923350	Treatment/medication code: co-dydramol	5.00E-06
20003_1140923402	Treatment/medication code: co-amilofruse	5.00E-08
20003_1140923484	Treatment/medication code: topiramate	5.00E-08
20003_1140923572	Treatment/medication code: adipine mr 10 m/r tablet	5.00E-08
20003_1140923670	Treatment/medication code: gtn - glyceryl trinitrate	5.00E-08
20003_1140923688	Treatment/medication code: zoton 15mg capsule	5.00E-08
20003_1140925800	Treatment/medication code: movicol oral powder	5.00E-08
20003_1140925978	Treatment/medication code: mycophenolate	5.00E-08
20003_1140926606	Treatment/medication code: salbutamol 100micrograms spacehaler	5.00E-06
20003_1140926732	Treatment/medication code: meloxicam	5.00E-08
20003_1140926780	Treatment/medication code: adizem-xl plus m/r capsule	5.00E-08
20003_1140926922	Treatment/medication code: antihistamine 60mg tablet	5.00E-08
20003_1140927086	Treatment/medication code: arthrotec 50 tablet	5.00E-08
20003_1140927320	Treatment/medication code: dermol 500 lotion	5.00E-08
20003_1140927328	Treatment/medication code: terbinafine	5.00E-08
20003_1140927730	Treatment/medication code: epaderm ointment	5.00E-08
20003_1140928004	Treatment/medication code: zimovane ls 3.75mg tablet	5.00E-08
20003_1140928274	Treatment/medication code: ropinirole	5.00E-08

Phenotype Code	Phenotype Description	Genome-wide Significance
20003_1140928284	Treatment/medication code: moxonidine	5.00E-08
20003_1140928916	Treatment/medication code: olanzapine	5.00E-08
20003_1140929012	Treatment/medication code: pantoprazole	5.00E-08
20003_1141145630	Treatment/medication code: nitromin 400micrograms cfc-free spray	5.00E-08
20003_1141145638	Treatment/medication code: nasobec aqueous 50micrograms nasal spray	5.00E-08
20003_1141145660	Treatment/medication code: valsartan	5.00E-06
20003_1141145668	Treatment/medication code: diovan 40mg capsule	5.00E-08
20003_1141145812	Treatment/medication code: minerals - magnesium	5.00E-08
20003_1141146138	Treatment/medication code: lipitor 10mg tablet	5.00E-06
20003_1141146188	Treatment/medication code: latanoprost	5.00E-08
20003_1141146198	Treatment/medication code: xalatan 0.005% eye drops	5.00E-08
20003_1141146234	Treatment/medication code: atorvastatin	5.00E-06
20003_1141146428	Treatment/medication code: fexofenadine	5.00E-08
20003_1141146508	Treatment/medication code: nasacort 55micrograms aqueous nasal spray	5.00E-08
20003_1141146606	Treatment/medication code: calcium carbonate+cholecalciferol 1.25g/200iu tablet	5.00E-08
20003_1141146612	Treatment/medication code: calceos chewable tablet	5.00E-08
20003_1141150620	Treatment/medication code: zolmitriptan	5.00E-08
20003_1141150624	Treatment/medication code: zomig 2.5mg tablet	5.00E-08
20003_1141150750	Treatment/medication code: brimonidine tartrate	5.00E-08
20003_1141150944	Treatment/medication code: nasonex 0.05% aqueous nasal spray	5.00E-06
20003_1141151016	Treatment/medication code: losartan potassium+hydrochlorothiazide 50mg/12.5mg tablet	5.00E-08
20003_1141151284	Treatment/medication code: naratriptan	5.00E-08
20003_1141151288	Treatment/medication code: naramig 2.5mg tablet	5.00E-08
20003_1141151946	Treatment/medication code: cipramil 10mg tablet	5.00E-08
20003_1141152590	Treatment/medication code: glimepiride	5.00E-08
20003_1141152732	Treatment/medication code: mirtazapine	5.00E-08
20003_1141152848	Treatment/medication code: quetiapine	5.00E-08
20003_1141152998	Treatment/medication code: irbesartan	5.00E-06
20003_1141153006	Treatment/medication code: aprovel 75mg tablet	5.00E-08
20003_1141153026	Treatment/medication code: lercanidipine	5.00E-08
20003_1141153032	Treatment/medication code: zanidip 10mg tablet	5.00E-08
20003_1141153242	Treatment/medication code: balsalazide disodium	5.00E-08
20003_1141156836	Treatment/medication code: candesartan cilexetil	5.00E-06
20003_1141156846	Treatment/medication code: amias 2mg tablet	5.00E-08
20003_1141157126	Treatment/medication code: montelukast product	5.00E-08
20003_1141157132	Treatment/medication code: singulair 10mg tablet	5.00E-08
20003_1141157178	Treatment/medication code: senna product	5.00E-08
20003_1141157252	Treatment/medication code: glyceryl trinitrate product	5.00E-08
20003_1141157264	Treatment/medication code: salmeterol product	5.00E-08
20003_1141157294	Treatment/medication code: hydrocortisone product	5.00E-08

Phenotype Code	Phenotype Description	Genome-wide Significance
20003_1141157402	Treatment/medication code: prednisolone product	5.00E-08
20003_1141157418	Treatment/medication code: budesonide product	5.00E-08
20003_1141157458	Treatment/medication code: hypromellose product	5.00E-08
20003_1141157486	Treatment/medication code: salbutamol product	5.00E-08
20003_1141157494	Treatment/medication code: ispaghula husk product	5.00E-08
20003_1141162764	Treatment/medication code: tolterodine l-tartrate	5.00E-08
20003_1141162824	Treatment/medication code: detrusitol 1mg tablet	5.00E-08
20003_1141164060	Treatment/medication code: pramipexole	5.00E-08
20003_1141164086	Treatment/medication code: salmeterol+fluticasone propionate	5.00E-08
20003_1141164276	Treatment/medication code: nebivolol	5.00E-08
20003_1141164828	Treatment/medication code: adcal-d3 1.5g/10micrograms chewable tablet	5.00E-06
20003_1141164872	Treatment/medication code: sinemet-62.5 tablet	5.00E-08
20003_1141166006	Treatment/medication code: telmisartan	5.00E-08
20003_1141166086	Treatment/medication code: rennie duo oral suspension	5.00E-08
20003_1141166294	Treatment/medication code: leflunomide	5.00E-08
20003_1141167334	Treatment/medication code: colofac-100 tablet	5.00E-08
20003_1141167594	Treatment/medication code: qvar 50 inhaler	5.00E-06
20003_1141167708	Treatment/medication code: beclomist 50micrograms nasal spray	5.00E-08
20003_1141167932	Treatment/medication code: rizatriptan	5.00E-08
20003_1141167940	Treatment/medication code: maxalt 5mg tablet	5.00E-08
20003_1141168108	Treatment/medication code: isotard 25xl m/r tablet	5.00E-08
20003_1141168122	Treatment/medication code: solpadol capsule	5.00E-08
20003_1141168318	Treatment/medication code: clopidogrel	5.00E-06
20003_1141168322	Treatment/medication code: plavix 75mg tablet	5.00E-08
20003_1141168554	Treatment/medication code: migraleve tablet	5.00E-08
20003_1141168584	Treatment/medication code: rabeprazole sodium	5.00E-08
20003_1141168590	Treatment/medication code: pariet 10mg e/c tablet	5.00E-08
20003_1141168680	Treatment/medication code: orlistat	5.00E-08
20003_1141168684	Treatment/medication code: xenical 120mg capsule	5.00E-08
20003_1141168752	Treatment/medication code: peptac liquid	5.00E-08
20003_1141169520	Treatment/medication code: cosopt 2%/0.5% eye drops	5.00E-08
20003_1141169844	Treatment/medication code: dexamethasone+hypromellose 0.1%/0.5% eye drops	5.00E-08
20003_1141171038	Treatment/medication code: oxycodone hydrochloride	5.00E-08
20003_1141171152	Treatment/medication code: cardicor 1.25mg tablet	5.00E-08
20003_1141171336	Treatment/medication code: eprosartan	5.00E-08
20003_1141171496	Treatment/medication code: trospium	5.00E-08
20003_1141171646	Treatment/medication code: pioglitazone	5.00E-08
20003_1141171932	Treatment/medication code: levetiracetam	5.00E-08
20003_1141171940	Treatment/medication code: keppra 250mg tablet	5.00E-08
20003_1141171948	Treatment/medication code: adcal 600mg chewable tablet	5.00E-06
20003_1141172492	Treatment/medication code: micardis 20mg tablet	5.00E-08

Phenotype Code	Phenotype Description	Genome-wide Significance
20003_1141172698	Treatment/medication code: monomax xl 60mg m/r tablet	5.00E-08
20003_1141172838	Treatment/medication code: depakote 250mg e/c tablet	5.00E-08
20003_1141172918	Treatment/medication code: celluvisc 1% single-use eye drops	5.00E-08
20003_1141172924	Treatment/medication code: desloratadine	5.00E-08
20003_1141172928	Treatment/medication code: neoclarityn 5mg tablet	5.00E-08
20003_1141173328	Treatment/medication code: ursodeoxycholic acid	5.00E-08
20003_1141173348	Treatment/medication code: hydroxocobalamin	5.00E-08
20003_1141174032	Treatment/medication code: doublebase gel	5.00E-08
20003_1141174508	Treatment/medication code: reductil 10mg capsule	5.00E-08
20003_1141174520	Treatment/medication code: symbicort 100/6 turbohaler	5.00E-06
20003_1141175684	Treatment/medication code: risedronate sodium	5.00E-08
20003_1141175690	Treatment/medication code: actonel 5mg tablet	5.00E-08
20003_1141176284	Treatment/medication code: brinzolamide	5.00E-08
20003_1141176288	Treatment/medication code: azopt 10mg/ml eye drops	5.00E-08
20003_1141176570	Treatment/medication code: fosamax 5mg tablet	5.00E-08
20003_1141176662	Treatment/medication code: celecoxib	5.00E-08
20003_1141176668	Treatment/medication code: celebrex 100mg capsule	5.00E-08
20003_1141176670	Treatment/medication code: celebrex 200mg capsule	5.00E-08
20003_1141176732	Treatment/medication code: carbomers	5.00E-08
20003_1141176832	Treatment/medication code: seretide 50 evohaler	5.00E-06
20003_1141177526	Treatment/medication code: esomeprazole	5.00E-06
20003_1141177532	Treatment/medication code: nexium 20mg tablet	5.00E-08
20003_1141177600	Treatment/medication code: rosiglitazone	5.00E-08
20003_1141178052	Treatment/medication code: zapain caplet	5.00E-08
20003_1141179764	Treatment/medication code: tenofovir	5.00E-08
20003_1141179914	Treatment/medication code: bimatoprost	5.00E-08
20003_1141179920	Treatment/medication code: lumigan 0.3mg/ml eye drops	5.00E-08
20003_1141179974	Treatment/medication code: cozaar 25mg tablet	5.00E-08
20003_1141179992	Treatment/medication code: dovobet ointment	5.00E-08
20003_1141180036	Treatment/medication code: fybogel orange s/f granules	5.00E-08
20003_1141180140	Treatment/medication code: etoricoxib	5.00E-08
20003_1141180148	Treatment/medication code: arcoxia 60mg tablet	5.00E-08
20003_1141180150	Treatment/medication code: arcoxia 90mg tablet	5.00E-08
20003_1141180212	Treatment/medication code: escitalopram	5.00E-08
20003_1141180226	Treatment/medication code: amoxicillin	5.00E-08
20003_1141180314	Treatment/medication code: ibandronic acid	5.00E-08
20003_1141180342	Treatment/medication code: beclometasone dipropionate+salbutamol	5.00E-08
20003_1141180392	Treatment/medication code: cefalexin	5.00E-08
20003_1141180662	Treatment/medication code: zirtek allergy 10mg tablet	5.00E-08
20003_1141180936	Treatment/medication code: calcium carbonate+colecalciferol 1.25g/5micrograms tablet	5.00E-08

Phenotype Code	Phenotype Description	Genome-wide Significance
20003_1141181868	Treatment/medication code: omacor 1g capsule	5.00E-08
20003_1141182628	Treatment/medication code: tiotropium	5.00E-08
20003_1141182632	Treatment/medication code: spiriva 18micrograms inhalation capsule	5.00E-08
20003_1141184726	Treatment/medication code: xalacom 0.005%/0.5% eye drops	5.00E-08
20003_1141184748	Treatment/medication code: levocetirizine	5.00E-08
20003_1141185316	Treatment/medication code: travoprost	5.00E-08
20003_1141185326	Treatment/medication code: travatan 40micrograms/ml eye drops	5.00E-08
20003_1141187230	Treatment/medication code: care cetirizine hayfever relief 10mg tablet	5.00E-08
20003_1141187776	Treatment/medication code: nurofen 200mg tablet	5.00E-08
20003_1141188146	Treatment/medication code: simvador 10mg tablet	5.00E-08
20003_1141188442	Treatment/medication code: glucosamine product	5.00E-06
20003_1141188594	Treatment/medication code: humira 40mg injection solution 0.8ml prefilled syringe	5.00E-08
20003_1141188658	Treatment/medication code: asacol mr 400mg e/c tablet	5.00E-08
20003_1141189090	Treatment/medication code: rosiglitazone 1mg / metformin 500mg tablet	5.00E-08
20003_1141189094	Treatment/medication code: avandamet 1mg / 500mg tablet	5.00E-08
20003_1141190158	Treatment/medication code: cipralex 5mg tablet	5.00E-08
20003_1141190160	Treatment/medication code: vascalpha 5mg m/r tablet	5.00E-08
20003_1141190960	Treatment/medication code: tramacet 325mg/37.5mg tablet	5.00E-08
20003_1141191044	Treatment/medication code: levothyroxine sodium	5.00E-06
20003_1141191748	Treatment/medication code: respiratory mometasone	5.00E-08
20003_1141192410	Treatment/medication code: rosuvastatin	5.00E-06
20003_1141192414	Treatment/medication code: crestor 10mg tablet	5.00E-08
20003_1141192736	Treatment/medication code: ezetimibe	5.00E-06
20003_1141192740	Treatment/medication code: ezetrol 10mg tablet	5.00E-08
20003_1141192916	Treatment/medication code: rino clenil 50micrograms nasal spray	5.00E-08
20003_1141193282	Treatment/medication code: olmesartan	5.00E-08
20003_1141193346	Treatment/medication code: olmetec 10mg tablet	5.00E-08
20003_1141194224	Treatment/medication code: allergy relief antihistamine 4mg tablet	5.00E-08
20003_1141194386	Treatment/medication code: telfast 30 tablet	5.00E-08
20003_1141194794	Treatment/medication code: bendroflumethiazide	5.00E-06
20003_1141195044	Treatment/medication code: hydroxycarbamide	5.00E-08
20003_1141195224	Treatment/medication code: formoterol	5.00E-08
20003_1141195232	Treatment/medication code: budesonide+formoterol	5.00E-08
20003_1141199858	Treatment/medication code: cardioplen xl 5mg m/r tablet	5.00E-08
20003_1141200004	Treatment/medication code: pregabalin	5.00E-08
20003_1141200072	Treatment/medication code: lyrica 25mg capsule	5.00E-08
20003_1141200108	Treatment/medication code: cymalon cranberry 1.5g/5ml liquid	5.00E-08
20003_1141200322	Treatment/medication code: solifenacin	5.00E-08
20003_1141200384	Treatment/medication code: vesicare 5mg tablet	5.00E-08
20003_1141200564	Treatment/medication code: duloxetine	5.00E-08
20003_1141200708	Treatment/medication code: strontium product	5.00E-08

Phenotype Code	Phenotype Description	Genome-wide Significance
20003_1141200768	Treatment/medication code: protelos 2g sachets	5.00E-08
20003_1187	Treatment/medication code: chondroitin product	5.00E-06
20003_1189	Treatment/medication code: co-enzyme q10/ubiquinone/bio-quinone/coenzyme q10	5.00E-08
20003_1193	Treatment/medication code: omega-3/fish oil supplement	5.00E-06
20003_1195	Treatment/medication code: vitamin c product	5.00E-08
20003_1197	Treatment/medication code: evening primrose oil product	5.00E-08
20003_1199	Treatment/medication code: food supplement/plant/herbal extract	5.00E-08
20003_1201	Treatment/medication code: st john's wort/hypericum [ctsu]	5.00E-08
20003_1203	Treatment/medication code: aloe vera product	5.00E-08
20003_2038459704	Treatment/medication code: carbamazepine	5.00E-08
20003_2038459814	Treatment/medication code: digoxin	5.00E-08
20003_2038460068	Treatment/medication code: phenobarbitone	5.00E-08
20003_2038460076	Treatment/medication code: phenytoin	5.00E-08
20003_2038460150	Treatment/medication code: paracetamol	5.00E-06
20003_99999	Treatment/medication code: Free-text entry, unable to be coded	5.00E-06

Table S2. Instrumental variables and associated parameters.

SNP	Effect_ allele	Other_ allele	Id. exposure	Beta. exposure	se. exposure	pval. exposure	eaf. exposure	Sample Size	R^2	R^2sum	F_statistics
rs113693316	A	G	Pregabalin	1.276E-02	2.262E-03	1.707E-08	6.374E-04	361141	8.806E-05	2.056E-03	33.821
rs116145451	C	T	Pregabalin	2.444E-03	4.057E-04	1.706E-09	1.989E-02	361141	1.005E-04		
rs140353506	A	G	Pregabalin	2.704E-03	4.805E-04	1.841E-08	1.570E-02	361141	8.765E-05		
rs140620527	T	C	Pregabalin	8.563E-03	1.555E-03	3.622E-08	1.368E-03	361141	8.401E-05		
rs143202005	A	T	Pregabalin	7.752E-03	1.401E-03	3.137E-08	1.823E-03	361141	8.479E-05		
rs143839978	C	G	Pregabalin	4.236E-03	7.632E-04	2.848E-08	5.780E-03	361141	8.530E-05		
rs183310363	C	G	Pregabalin	8.420E-03	1.513E-03	2.595E-08	1.360E-03	361141	8.581E-05		
rs183953713	G	C	Pregabalin	8.302E-03	1.431E-03	6.515E-09	1.671E-03	361141	9.324E-05		
rs184678115	A	C	Pregabalin	4.794E-03	8.790E-04	4.934E-08	4.373E-03	361141	8.235E-05		
rs187715970	A	G	Pregabalin	9.913E-03	1.694E-03	4.875E-09	1.213E-03	361141	9.480E-05		
rs190996522	T	C	Pregabalin	6.167E-03	1.097E-03	1.912E-08	2.609E-03	361141	8.745E-05		
rs191878997	A	G	Pregabalin	1.035E-02	1.614E-03	1.445E-10	1.155E-03	361141	1.138E-04		
rs192127317	T	G	Pregabalin	8.436E-03	1.481E-03	1.233E-08	1.603E-03	361141	8.981E-05		
rs527410274	G	T	Pregabalin	7.214E-03	1.130E-03	1.730E-10	2.719E-03	361141	1.128E-04		
rs530199139	C	A	Pregabalin	7.572E-03	1.354E-03	2.232E-08	1.926E-03	361141	8.662E-05		
rs538098668	T	C	Pregabalin	8.022E-03	1.276E-03	3.249E-10	1.920E-03	361141	1.094E-04		
rs539226356	C	A	Pregabalin	9.766E-03	1.761E-03	2.903E-08	1.098E-03	361141	8.520E-05		
rs544834902	A	G	Pregabalin	7.362E-03	1.240E-03	2.899E-09	2.269E-03	361141	9.760E-05		
rs547838929	T	C	Pregabalin	9.207E-03	1.659E-03	2.859E-08	1.226E-03	361141	8.528E-05		
rs554861870	C	T	Pregabalin	4.729E-03	8.292E-04	1.173E-08	4.727E-03	361141	9.007E-05		
rs568747619	A	G	Pregabalin	7.601E-03	1.145E-03	3.156E-11	2.381E-03	361141	1.220E-04		
rs569634086	A	G	Pregabalin	9.067E-03	1.594E-03	1.279E-08	1.357E-03	361141	8.961E-05		
rs115727531	A	G	Bumetanide	2.467E-03	4.453E-04	3.035E-08	6.209E-03	361141	8.496E-05	7.326E-03	39.189
rs116886112	A	T	Bumetanide	1.362E-03	2.488E-04	4.371E-08	1.982E-02	361141	8.300E-05		
rs117695801	T	C	Bumetanide	5.258E-03	9.224E-04	1.200E-08	1.641E-03	361141	8.995E-05		
rs118070463	C	T	Bumetanide	3.523E-03	5.891E-04	2.225E-09	3.508E-03	361141	9.903E-05		
rs11879837	C	A	Bumetanide	6.225E-03	8.494E-04	2.321E-13	2.654E-03	361141	1.487E-04		
rs138139611	T	C	Bumetanide	3.405E-02	5.406E-03	3.023E-10	4.342E-05	361141	1.098E-04		
rs138554630	T	C	Bumetanide	1.591E-03	2.632E-04	1.500E-09	1.807E-02	361141	1.012E-04		
rs139815340	A	G	Bumetanide	1.369E-02	2.462E-03	2.689E-08	2.118E-04	361141	8.561E-05		
rs141528208	C	T	Bumetanide	4.400E-03	6.785E-04	8.860E-11	2.651E-03	361141	1.165E-04		
rs142578249	C	T	Bumetanide	5.961E-03	1.061E-03	1.954E-08	1.022E-03	361141	8.733E-05		
rs143135288	G	T	Bumetanide	1.442E-02	1.780E-03	5.531E-16	3.627E-04	361141	1.816E-04		
rs143485240	T	C	Bumetanide	5.386E-03	9.342E-04	8.161E-09	1.512E-03	361141	9.203E-05		
rs145622607	T	C	Bumetanide	5.733E-03	9.121E-04	3.261E-10	1.588E-03	361141	1.094E-04		
rs145740954	C	G	Bumetanide	6.318E-03	9.740E-04	8.768E-11	1.371E-03	361141	1.165E-04		
rs145958332	T	C	Bumetanide	6.021E-03	9.211E-04	6.276E-11	1.556E-03	361141	1.183E-04		
rs146783569	A	G	Bumetanide	5.423E-03	7.826E-04	4.225E-12	2.246E-03	361141	1.330E-04		
rs146963758	A	G	Bumetanide	2.647E-02	2.394E-03	2.060E-28	2.331E-04	361141	3.384E-04		
rs147204866	T	C	Bumetanide	4.197E-03	7.372E-04	1.248E-08	2.535E-03	361141	8.974E-05		
rs149647118	C	G	Bumetanide	3.088E-03	5.313E-04	6.184E-09	4.340E-03	361141	9.352E-05		
rs181737245	A	G	Bumetanide	8.425E-03	1.125E-03	6.847E-14	1.080E-03	361141	1.554E-04		
rs182207603	T	A	Bumetanide	2.889E-03	5.273E-04	4.274E-08	4.600E-03	361141	8.312E-05		
rs182408184	T	C	Bumetanide	5.629E-03	9.585E-04	4.283E-09	1.409E-03	361141	9.550E-05		
rs184816120	T	C	Bumetanide	4.135E-03	7.274E-04	1.310E-08	2.567E-03	361141	8.948E-05		
rs185170930	A	G	Bumetanide	5.462E-03	8.145E-04	2.015E-11	2.033E-03	361141	1.245E-04		
rs185206583	G	T	Bumetanide	5.413E-03	9.350E-04	7.102E-09	1.442E-03	361141	9.278E-05		
rs186430919	T	C	Bumetanide	7.906E-03	1.107E-03	9.270E-13	9.900E-04	361141	1.412E-04		

SNP	Effect_ allele	Other_ allele	Id. exposure	Beta. exposure	se. exposure	pval. exposure	eaf. exposure	Sample Size	R^2	R^2sum	*F*_statistics
rs186663269	T	C	Bumetanide	5.338E-03	8.627E-04	6.104E-10	1.747E-03	361141	1.060E-04		
rs186992246	T	G	Bumetanide	3.997E-03	6.768E-04	3.499E-09	2.883E-03	361141	9.659E-05		
rs187532190	T	C	Bumetanide	3.744E-03	6.537E-04	1.021E-08	2.962E-03	361141	9.082E-05		
rs187703469	A	G	Bumetanide	3.229E-03	5.453E-04	3.193E-09	4.215E-03	361141	9.708E-05		
rs188351362	T	C	Bumetanide	5.742E-03	1.049E-03	4.399E-08	1.088E-03	361141	8.297E-05		
rs188931867	T	G	Bumetanide	5.321E-03	9.430E-04	1.679E-08	1.354E-03	361141	8.815E-05		
rs189287152	T	G	Bumetanide	2.943E-03	4.885E-04	1.708E-09	5.436E-03	361141	1.005E-04		
rs189924545	G	A	Bumetanide	3.972E-03	7.284E-04	4.952E-08	2.366E-03	361141	8.233E-05		
rs190028005	C	T	Bumetanide	6.212E-03	9.865E-04	3.034E-10	1.408E-03	361141	1.098E-04		
rs192240983	A	G	Bumetanide	4.187E-03	6.763E-04	6.003E-10	2.807E-03	361141	1.061E-04		
rs192651543	C	T	Bumetanide	4.422E-03	8.012E-04	3.395E-08	2.076E-03	361141	8.436E-05		
rs192755009	T	C	Bumetanide	7.211E-03	1.080E-03	2.454E-11	1.125E-03	361141	1.234E-04		
rs193158986	T	C	Bumetanide	5.875E-03	1.042E-03	1.742E-08	1.145E-03	361141	8.795E-05		
rs202084316	A	G	Bumetanide	7.968E-02	8.246E-03	4.333E-22	2.074E-05	361141	2.585E-04		
rs34909006	C	G	Bumetanide	4.261E-03	6.892E-04	6.313E-10	2.590E-03	361141	1.058E-04		
rs367877683	T	C	Bumetanide	5.917E-03	9.709E-04	1.104E-09	1.345E-03	361141	1.028E-04		
rs370715182	C	T	Bumetanide	5.371E-03	9.494E-04	1.542E-08	1.437E-03	361141	8.860E-05		
rs3770319	T	A	Bumetanide	5.915E-03	9.038E-04	5.983E-11	1.540E-03	361141	1.186E-04		
rs41476246	T	C	Bumetanide	2.008E-02	2.995E-03	2.050E-11	2.286E-04	361141	1.244E-04		
rs527305967	A	G	Bumetanide	5.128E-03	9.301E-04	3.523E-08	1.475E-03	361141	8.416E-05		
rs527506011	C	T	Bumetanide	6.880E-03	1.150E-03	2.186E-09	9.623E-04	361141	9.913E-05		
rs527614517	A	G	Bumetanide	5.992E-03	1.072E-03	2.302E-08	1.097E-03	361141	8.645E-05		
rs529837198	T	C	Bumetanide	5.955E-03	1.036E-03	8.919E-09	1.157E-03	361141	9.155E-05		
rs538401172	T	C	Bumetanide	5.292E-03	9.517E-04	2.682E-08	1.509E-03	361141	8.563E-05		
rs541275209	C	T	Bumetanide	5.281E-03	9.427E-04	2.117E-08	1.439E-03	361141	8.690E-05		
rs551191688	A	G	Bumetanide	4.608E-03	6.784E-04	1.097E-11	2.688E-03	361141	1.278E-04		
rs552621692	T	G	Bumetanide	4.981E-03	8.316E-04	2.106E-09	1.777E-03	361141	9.933E-05		
rs553068214	A	T	Bumetanide	3.724E-03	6.447E-04	7.659E-09	2.952E-03	361141	9.237E-05		
rs55956854	T	A	Bumetanide	1.198E-03	1.932E-04	5.540E-10	3.309E-02	361141	1.065E-04		
rs562184820	A	C	Bumetanide	6.670E-03	1.098E-03	1.225E-09	1.165E-03	361141	1.023E-04		
rs567822095	A	T	Bumetanide	4.979E-03	9.004E-04	3.204E-08	1.622E-03	361141	8.467E-05		
rs568079472	A	G	Bumetanide	5.447E-03	9.978E-04	4.791E-08	1.410E-03	361141	8.251E-05		
rs575169528	A	G	Bumetanide	4.896E-03	8.465E-04	7.280E-09	1.748E-03	361141	9.264E-05		
rs59079584	T	C	Bumetanide	2.157E-02	3.036E-03	1.216E-12	1.246E-04	361141	1.397E-04		
rs72752095	C	T	Bumetanide	4.411E-03	7.817E-04	1.670E-08	2.337E-03	361141	8.817E-05		
rs73012938	A	C	Bumetanide	6.207E-03	1.003E-03	6.060E-10	1.383E-03	361141	1.061E-04		
rs74349775	C	T	Bumetanide	6.071E-03	9.545E-04	2.013E-10	1.547E-03	361141	1.120E-04		
rs7593869	C	T	Bumetanide	2.746E-03	4.677E-04	4.333E-09	5.847E-03	361141	9.544E-05		
rs76526509	C	G	Bumetanide	4.064E-03	7.381E-04	3.684E-08	2.221E-03	361141	8.392E-05		
rs77285146	T	C	Bumetanide	6.423E-03	1.100E-03	5.287E-09	1.034E-03	361141	9.437E-05		
rs79003258	T	C	Bumetanide	1.132E-03	2.046E-04	3.088E-08	2.827E-02	361141	8.487E-05		
rs7991670	T	C	Bumetanide	1.726E-03	3.121E-04	3.213E-08	1.199E-02	361141	8.466E-05		
rs11058355	A	G	Prednisolone	1.279E-03	2.737E-04	2.994E-06	1.226E-01	361141	6.042E-05	5.489E-03	25.227
rs114305799	C	T	Prednisolone	5.597E-02	1.093E-02	3.064E-07	6.790E-05	361141	7.257E-05		
rs114441734	T	C	Prednisolone	5.775E-03	1.212E-03	1.909E-06	5.628E-03	361141	6.281E-05		
rs117599532	A	T	Prednisolone	4.248E-03	9.232E-04	4.202E-06	1.022E-02	361141	5.862E-05		
rs12973314	C	T	Prednisolone	-1.075E-03	2.281E-04	2.442E-06	2.044E-01	361141	6.150E-05		
rs139365458	G	T	Prednisolone	7.930E-03	1.630E-03	1.149E-06	3.680E-03	361141	6.552E-05		
rs139507384	G	T	Prednisolone	6.220E-03	1.337E-03	3.293E-06	5.026E-03	361141	5.991E-05		
rs140100052	A	C	Prednisolone	8.183E-03	1.593E-03	2.804E-07	3.563E-03	361141	7.304E-05		

SNP	Effect_ allele	Other_ allele	Id. exposure	Beta. exposure	se. exposure	pval. exposure	eaf. exposure	Sample Size	R^2	R^2sum	*F*_statistics
rs141526402	T	A	Prednisolone	3.728E-02	7.888E-03	2.282E-06	1.288E-04	361141	6.186E-05		
rs141840050	C	T	Prednisolone	1.008E-02	1.940E-03	2.037E-07	2.228E-03	361141	7.475E-05		
rs141945040	A	G	Prednisolone	1.025E-02	2.244E-03	4.880E-06	1.878E-03	361141	5.783E-05		
rs142129661	A	G	Prednisolone	9.124E-03	1.882E-03	1.248E-06	2.619E-03	361141	6.508E-05		
rs142808011	T	C	Prednisolone	8.139E-03	1.548E-03	1.467E-07	4.090E-03	361141	7.651E-05		
rs142913425	T	C	Prednisolone	9.407E-03	1.783E-03	1.330E-07	3.098E-03	361141	7.704E-05		
rs144246060	C	T	Prednisolone	1.187E-02	2.471E-03	1.572E-06	1.542E-03	361141	6.385E-05		
rs144606442	T	C	Prednisolone	7.502E-03	1.485E-03	4.378E-07	4.348E-03	361141	7.066E-05		
rs144702811	T	C	Prednisolone	1.254E-02	2.529E-03	7.176E-07	1.408E-03	361141	6.802E-05		
rs144821073	A	G	Prednisolone	4.894E-03	1.041E-03	2.600E-06	8.101E-03	361141	6.117E-05		
rs145175533	C	T	Prednisolone	1.029E-02	2.104E-03	1.012E-06	1.903E-03	361141	6.619E-05		
rs145944200	G	A	Prednisolone	6.734E-03	1.439E-03	2.889E-06	4.313E-03	361141	6.061E-05		
rs146410697	T	C	Prednisolone	8.670E-03	1.897E-03	4.837E-06	2.625E-03	361141	5.787E-05		
rs148449869	G	T	Prednisolone	1.117E-02	2.297E-03	1.153E-06	1.847E-03	361141	6.550E-05		
rs148800348	T	C	Prednisolone	1.486E-02	2.951E-03	4.786E-07	9.959E-04	361141	7.019E-05		
rs148837974	A	G	Prednisolone	8.405E-03	1.604E-03	1.597E-07	3.705E-03	361141	7.606E-05		
rs149035807	G	A	Prednisolone	3.418E-03	7.314E-04	2.963E-06	1.619E-02	361141	6.047E-05		
rs150440298	A	G	Prednisolone	1.786E-02	3.853E-03	3.550E-06	6.353E-04	361141	5.951E-05		
rs151090482	A	C	Prednisolone	4.470E-03	9.474E-04	2.379E-06	1.022E-02	361141	6.164E-05		
rs181363103	C	T	Prednisolone	9.942E-03	2.080E-03	1.765E-06	2.265E-03	361141	6.323E-05		
rs182093009	C	A	Prednisolone	8.064E-03	1.747E-03	3.926E-06	3.079E-03	361141	5.898E-05		
rs182513921	A	T	Prednisolone	1.326E-02	2.841E-03	3.056E-06	1.216E-03	361141	6.031E-05		
rs182781691	A	C	Prednisolone	1.422E-02	2.744E-03	2.203E-07	1.178E-03	361141	7.434E-05		
rs184462623	T	G	Prednisolone	1.175E-02	2.482E-03	2.178E-06	1.567E-03	361141	6.211E-05		
rs185422815	C	A	Prednisolone	1.529E-02	3.113E-03	8.930E-07	9.413E-04	361141	6.686E-05		
rs186287119	G	C	Prednisolone	8.600E-03	1.775E-03	1.259E-06	2.796E-03	361141	6.503E-05		
rs186409181	T	C	Prednisolone	7.113E-03	1.327E-03	8.362E-08	5.538E-03	361141	7.952E-05		
rs186624402	A	T	Prednisolone	1.005E-02	2.100E-03	1.709E-06	1.968E-03	361141	6.340E-05		
rs187360712	T	C	Prednisolone	8.560E-03	1.856E-03	3.988E-06	2.594E-03	361141	5.890E-05		
rs188575117	C	A	Prednisolone	2.257E-03	2.454E-04	3.609E-20	1.728E-01	361141	2.343E-04		
rs188626859	C	T	Prednisolone	6.284E-03	1.183E-03	1.088E-07	6.160E-03	361141	7.811E-05		
rs188640222	C	A	Prednisolone	1.265E-02	2.572E-03	8.835E-07	1.502E-03	361141	6.692E-05		
rs188914868	T	G	Prednisolone	9.372E-03	1.971E-03	1.981E-06	2.272E-03	361141	6.261E-05		
rs189686022	A	G	Prednisolone	7.889E-03	1.661E-03	2.053E-06	3.271E-03	361141	6.243E-05		
rs191884665	G	C	Prednisolone	8.242E-03	1.756E-03	2.698E-06	2.712E-03	361141	6.097E-05		
rs191923166	A	G	Prednisolone	1.304E-02	2.203E-03	3.295E-09	2.000E-03	361141	9.691E-05		
rs191979014	G	T	Prednisolone	7.131E-03	1.485E-03	1.579E-06	3.944E-03	361141	6.382E-05		
rs192314281	C	T	Prednisolone	8.192E-03	1.778E-03	4.094E-06	2.759E-03	361141	5.876E-05		
rs192335405	A	G	Prednisolone	7.942E-03	1.496E-03	1.100E-07	4.413E-03	361141	7.806E-05		
rs192702494	A	G	Prednisolone	6.121E-03	1.308E-03	2.862E-06	5.447E-03	361141	6.066E-05		
rs192827646	A	G	Prednisolone	1.551E-02	2.813E-03	3.518E-08	1.181E-03	361141	8.417E-05		
rs235162	C	C	Prednisolone	-1.198E-03	2.621E-04	4.822E-06	1.394E-01	361141	5.789E-05		
rs2471960	A	G	Prednisolone	1.046E-03	1.935E-04	6.464E-08	3.122E-01	361141	8.090E-05		
rs2476601	A	A	Prednisolone	-1.426E-03	2.948E-04	1.324E-06	1.024E-01	361141	6.476E-05		
rs326480	T	C	Prednisolone	9.320E-04	1.927E-04	1.318E-06	3.163E-01	361141	6.478E-05		
rs35106972	C	G	Prednisolone	5.374E-03	1.096E-03	9.324E-07	7.244E-03	361141	6.663E-05		
rs35511257	C	G	Prednisolone	1.648E-03	3.268E-04	4.586E-07	9.515E-02	361141	7.041E-05		
rs3791327	G	C	Prednisolone	-1.467E-03	2.954E-04	6.837E-07	1.057E-01	361141	6.828E-05		
rs528966891	A	G	Prednisolone	1.380E-02	2.884E-03	1.698E-06	1.095E-03	361141	6.343E-05		
rs536120076	G	A	Prednisolone	1.613E-02	2.737E-03	3.793E-09	1.313E-03	361141	9.616E-05		
rs537871790	A	G	Prednisolone	1.035E-02	2.048E-03	4.312E-07	2.229E-03	361141	7.074E-05		

SNP	Effect_ allele	Other_ allele	Id. exposure	Beta. exposure	se. exposure	pval. exposure	eaf. exposure	Sample Size	R^2	R^2sum	*F*_statistics
rs537933035	T	G	Prednisolone	9.930E-03	2.107E-03	2.431E-06	2.149E-03	361141	6.152E-05		
rs540356727	G	A	Prednisolone	1.285E-02	2.665E-03	1.428E-06	1.376E-03	361141	6.436E-05		
rs544819271	T	C	Prednisolone	1.526E-02	2.794E-03	4.717E-08	1.242E-03	361141	8.260E-05		
rs549295739	T	C	Prednisolone	2.972E-02	5.894E-03	4.595E-07	2.741E-04	361141	7.040E-05		
rs549425051	C	T	Prednisolone	6.740E-03	1.417E-03	1.985E-06	4.905E-03	361141	6.260E-05		
rs556846474	T	C	Prednisolone	1.291E-02	2.683E-03	1.496E-06	1.316E-03	361141	6.411E-05		
rs56028730	C	T	Prednisolone	2.155E-02	4.715E-03	4.861E-06	4.091E-04	361141	5.785E-05		
rs561002768	T	C	Prednisolone	8.421E-03	1.734E-03	1.191E-06	3.081E-03	361141	6.532E-05		
rs565616076	C	G	Prednisolone	7.359E-03	1.611E-03	4.892E-06	3.452E-03	361141	5.781E-05		
rs572210321	C	T	Prednisolone	1.203E-02	2.301E-03	1.730E-07	1.763E-03	361141	7.563E-05		
rs573164860	A	G	Prednisolone	1.037E-02	2.245E-03	3.837E-06	1.804E-03	361141	5.910E-05		
rs573367732	T	C	Prednisolone	6.989E-03	1.420E-03	8.562E-07	4.314E-03	361141	6.708E-05		
rs575851300	T	A	Prednisolone	1.353E-02	2.538E-03	9.827E-08	1.415E-03	361141	7.866E-05		
rs577351994	A	T	Prednisolone	1.047E-02	2.088E-03	5.281E-07	2.097E-03	361141	6.966E-05		
rs72755615	C	T	Prednisolone	7.102E-03	1.271E-03	2.308E-08	5.825E-03	361141	8.643E-05		
rs73085068	A	G	Prednisolone	5.093E-03	1.007E-03	4.282E-07	8.054E-03	361141	7.078E-05		
rs74940988	C	T	Prednisolone	-2.713E-03	5.180E-04	1.624E-07	3.090E-02	361141	7.597E-05		
rs75251667	T	C	Prednisolone	1.014E-02	2.176E-03	3.150E-06	1.906E-03	361141	6.015E-05		
rs76255908	G	T	Prednisolone	7.117E-03	1.476E-03	1.415E-06	3.961E-03	361141	6.441E-05		
rs79560480	A	G	Prednisolone	4.187E-03	8.900E-04	2.553E-06	1.022E-02	361141	6.126E-05		
rs10976069	T	C	Vitamin b1 preparation	3.963E-03	6.155E-04	1.198E-10	1.781E-03	361141	1.148E-04	1.638E-02	38.278
rs112433664	A	G	Vitamin b1 preparation	3.284E-03	5.956E-04	3.510E-08	2.042E-03	361141	8.418E-05		
rs112577881	C	T	Vitamin b1 preparation	2.584E-03	4.469E-04	7.414E-09	3.239E-03	361141	9.254E-05		
rs114696482	A	G	Vitamin b1 preparation	1.719E-03	2.843E-04	1.493E-09	8.429E-03	361141	1.012E-04		
rs114778612	A	T	Vitamin b1 preparation	2.461E-03	4.308E-04	1.117E-08	3.920E-03	361141	9.034E-05		
rs115201875	C	G	Vitamin b1 preparation	2.445E-02	4.420E-03	3.146E-08	7.473E-05	361141	8.477E-05		
rs116427224	A	C	Vitamin b1 preparation	2.627E-02	3.406E-03	1.219E-14	6.630E-05	361141	1.648E-04		
rs116981543	C	T	Vitamin b1 preparation	9.835E-03	1.526E-03	1.162E-10	2.716E-04	361141	1.150E-04		
rs117985603	T	C	Vitamin b1 preparation	2.985E-03	4.773E-04	4.049E-10	3.129E-03	361141	1.082E-04		
rs118146661	T	C	Vitamin b1 preparation	2.614E-03	3.905E-04	2.172E-11	4.477E-03	361141	1.241E-04		
rs12497303	G	A	Vitamin b1 preparation	5.055E-03	7.758E-04	7.246E-11	1.094E-03	361141	1.175E-04		
rs12615630	T	T	Vitamin b1 preparation	-8.049E-04	1.469E-04	4.251E-08	2.866E-02	361141	8.315E-05		
rs138238661	T	C	Vitamin b1 preparation	2.815E-03	4.294E-04	5.565E-11	3.566E-03	361141	1.190E-04		
rs138537851	C	T	Vitamin b1 preparation	3.593E-03	6.578E-04	4.712E-08	1.640E-03	361141	8.260E-05		
rs139512326	C	T	Vitamin b1 preparation	4.391E-03	6.472E-04	1.161E-11	1.463E-03	361141	1.275E-04		
rs139637641	T	C	Vitamin b1 preparation	3.063E-03	5.122E-04	2.223E-09	2.559E-03	361141	9.904E-05		
rs140547617	C	G	Vitamin b1 preparation	2.211E-03	3.886E-04	1.274E-08	4.199E-03	361141	8.963E-05		
rs140960245	T	C	Vitamin b1 preparation	2.320E-03	3.871E-04	2.071E-09	4.175E-03	361141	9.942E-05		
rs141380913	G	T	Vitamin b1 preparation	2.451E-03	4.411E-04	2.748E-08	3.631E-03	361141	8.550E-05		
rs142052690	G	T	Vitamin b1 preparation	2.387E-03	4.247E-04	1.911E-08	3.899E-03	361141	8.745E-05		
rs142257574	A	G	Vitamin b1 preparation	4.132E-03	6.751E-04	9.357E-10	1.458E-03	361141	1.037E-04		

SNP	Effect_ allele	Other_ allele	Id. exposure	Beta. exposure	se. exposure	pval. exposure	eaf. exposure	Sample Size	R^2	R^2sum	F_statistics
rs142351283	C	A	Vitamin b1 preparation	1.961E-03	3.515E-04	2.437E-08	5.334E-03	361141	8.614E-05		
rs142409803	C	T	Vitamin b1 preparation	1.263E-02	1.722E-03	2.219E-13	2.433E-04	361141	1.490E-04		
rs142489686	C	T	Vitamin b1 preparation	2.928E-03	5.145E-04	1.263E-08	2.603E-03	361141	8.968E-05		
rs143248101	A	C	Vitamin b1 preparation	3.367E-03	5.901E-04	1.161E-08	1.923E-03	361141	9.013E-05		
rs143416612	A	G	Vitamin b1 preparation	4.267E-03	7.190E-04	2.946E-09	1.355E-03	361141	9.752E-05		
rs145115245	C	T	Vitamin b1 preparation	1.648E-03	2.936E-04	1.999E-08	8.683E-03	361141	8.721E-05		
rs145122245	G	A	Vitamin b1 preparation	1.781E-03	3.156E-04	1.665E-08	7.339E-03	361141	8.819E-05		
rs145580153	G	A	Vitamin b1 preparation	3.516E-03	5.538E-04	2.158E-10	2.363E-03	361141	1.116E-04		
rs145796458	C	T	Vitamin b1 preparation	2.490E-03	4.466E-04	2.449E-08	3.653E-03	361141	8.612E-05		
rs145830894	T	C	Vitamin b1 preparation	2.016E-03	3.404E-04	3.162E-09	6.322E-03	361141	9.714E-05		
rs146536518	C	T	Vitamin b1 preparation	3.968E-03	6.368E-04	4.640E-10	1.650E-03	361141	1.075E-04		
rs146694363	G	A	Vitamin b1 preparation	4.321E-03	7.187E-04	1.838E-09	1.270E-03	361141	1.001E-04		
rs147263320	T	C	Vitamin b1 preparation	8.788E-03	1.411E-03	4.733E-10	3.004E-04	361141	1.074E-04		
rs147503987	G	A	Vitamin b1 preparation	3.902E-03	6.638E-04	4.144E-09	1.498E-03	361141	9.568E-05		
rs147931408	T	C	Vitamin b1 preparation	2.932E-03	5.039E-04	5.972E-09	2.831E-03	361141	9.371E-05		
rs148356236	G	A	Vitamin b1 preparation	4.336E-02	4.777E-03	1.130E-19	3.132E-05	361141	2.280E-04		
rs149122000	T	C	Vitamin b1 preparation	1.983E-02	2.965E-03	2.288E-11	7.878E-05	361141	1.238E-04		
rs149192078	G	A	Vitamin b1 preparation	4.700E-03	7.253E-04	9.158E-11	1.352E-03	361141	1.163E-04		
rs150039604	A	G	Vitamin b1 preparation	2.896E-03	5.229E-04	3.046E-08	2.598E-03	361141	8.494E-05		
rs150103511	A	G	Vitamin b1 preparation	3.131E-03	5.687E-04	3.695E-08	2.075E-03	361141	8.391E-05		
rs150116102	C	T	Vitamin b1 preparation	6.192E-03	9.769E-04	2.318E-10	6.244E-04	361141	1.112E-04		
rs150542170	T	C	Vitamin b1 preparation	5.514E-03	7.392E-04	8.695E-14	1.319E-03	361141	1.541E-04		
rs151168294	C	T	Vitamin b1 preparation	2.290E-03	4.180E-04	4.296E-08	3.928E-03	361141	8.310E-05		
rs180919900	T	G	Vitamin b1 preparation	3.903E-03	7.055E-04	3.168E-08	1.418E-03	361141	8.473E-05		
rs180928644	A	G	Vitamin b1 preparation	2.143E-03	3.535E-04	1.358E-09	5.285E-03	361141	1.017E-04		
rs181204764	G	A	Vitamin b1 preparation	2.457E-03	4.038E-04	1.161E-09	4.215E-03	361141	1.025E-04		
rs181269977	G	A	Vitamin b1 preparation	4.671E-03	7.262E-04	1.258E-10	1.372E-03	361141	1.146E-04		
rs181374026	T	C	Vitamin b1 preparation	4.044E-03	5.877E-04	5.978E-12	2.025E-03	361141	1.311E-04		
rs181930675	A	G	Vitamin b1 preparation	3.559E-03	5.358E-04	3.090E-11	2.408E-03	361141	1.222E-04		
rs182816978	G	A	Vitamin b1 preparation	5.171E-03	7.579E-04	8.981E-12	1.258E-03	361141	1.289E-04		
rs182853670	T	C	Vitamin b1 preparation	2.659E-03	4.617E-04	8.458E-09	3.270E-03	361141	9.183E-05		
rs183138930	T	G	Vitamin b1 preparation	3.467E-03	5.898E-04	4.170E-09	2.034E-03	361141	9.565E-05		
rs183252511	C	G	Vitamin b1 preparation	4.127E-03	6.794E-04	1.249E-09	1.505E-03	361141	1.021E-04		
rs183255252	T	C	Vitamin b1 preparation	3.709E-03	6.631E-04	2.233E-08	1.600E-03	361141	8.661E-05		
rs183332202	C	T	Vitamin b1 preparation	1.999E-03	3.477E-04	8.991E-09	5.730E-03	361141	9.151E-05		

SNP	Effect_ allele	Other_ allele	Id. exposure	Beta. exposure	se. exposure	pval. exposure	eaf. exposure	Sample Size	R^2	R^2sum	F_statistics
rs183442754	A	G	Vitamin b1 preparation	3.236E-03	5.842E-04	3.033E-08	1.984E-03	361141	8.497E-05		
rs183611846	T	C	Vitamin b1 preparation	3.934E-03	6.254E-04	3.153E-10	1.753E-03	361141	1.096E-04		
rs183763481	T	C	Vitamin b1 preparation	2.675E-03	4.901E-04	4.830E-08	2.794E-03	361141	8.247E-05		
rs183779241	G	A	Vitamin b1 preparation	2.944E-03	4.446E-04	3.575E-11	3.491E-03	361141	1.214E-04		
rs184105029	T	A	Vitamin b1 preparation	2.246E-03	3.724E-04	1.618E-09	4.760E-03	361141	1.007E-04		
rs184144024	T	G	Vitamin b1 preparation	2.147E-03	3.800E-04	1.611E-08	4.608E-03	361141	8.837E-05		
rs184161070	C	A	Vitamin b1 preparation	3.692E-03	6.391E-04	7.607E-09	1.777E-03	361141	9.241E-05		
rs184336995	A	T	Vitamin b1 preparation	2.453E-03	4.330E-04	1.481E-08	3.749E-03	361141	8.882E-05		
rs184503364	T	A	Vitamin b1 preparation	3.354E-03	5.134E-04	6.443E-11	2.604E-03	361141	1.182E-04		
rs184769175	A	G	Vitamin b1 preparation	2.679E-03	4.912E-04	4.926E-08	2.749E-03	361141	8.236E-05		
rs185056177	T	C	Vitamin b1 preparation	4.464E-03	7.331E-04	1.131E-09	1.259E-03	361141	1.027E-04		
rs185564439	T	G	Vitamin b1 preparation	3.646E-03	5.575E-04	6.209E-11	2.146E-03	361141	1.184E-04		
rs185840944	A	G	Vitamin b1 preparation	3.266E-03	5.899E-04	3.081E-08	1.868E-03	361141	8.488E-05		
rs185911369	A	G	Vitamin b1 preparation	2.649E-03	4.642E-04	1.149E-08	3.145E-03	361141	9.019E-05		
rs186253228	C	A	Vitamin b1 preparation	2.444E-03	4.006E-04	1.056E-09	4.139E-03	361141	1.031E-04		
rs186287265	A	G	Vitamin b1 preparation	3.913E-03	6.905E-04	1.455E-08	1.366E-03	361141	8.892E-05		
rs186396877	T	C	Vitamin b1 preparation	3.848E-03	5.756E-04	2.300E-11	1.949E-03	361141	1.238E-04		
rs186920839	C	T	Vitamin b1 preparation	1.950E-03	3.345E-04	5.562E-09	5.913E-03	361141	9.409E-05		
rs187384616	T	C	Vitamin b1 preparation	2.946E-03	5.201E-04	1.471E-08	2.671E-03	361141	8.886E-05		
rs187521595	T	C	Vitamin b1 preparation	4.425E-03	6.983E-04	2.339E-10	1.463E-03	361141	1.112E-04		
rs187726921	G	A	Vitamin b1 preparation	2.599E-03	4.755E-04	4.625E-08	2.880E-03	361141	8.270E-05		
rs187863876	G	A	Vitamin b1 preparation	4.639E-03	6.802E-04	9.090E-12	1.503E-03	361141	1.288E-04		
rs187916922	C	G	Vitamin b1 preparation	4.044E-03	6.807E-04	2.841E-09	1.545E-03	361141	9.771E-05		
rs188083604	T	C	Vitamin b1 preparation	1.835E-03	3.329E-04	3.575E-08	6.565E-03	361141	8.408E-05		
rs188856748	C	A	Vitamin b1 preparation	4.338E-03	7.582E-04	1.057E-08	1.269E-03	361141	9.063E-05		
rs189159984	T	C	Vitamin b1 preparation	3.365E-03	5.498E-04	9.409E-10	2.424E-03	361141	1.037E-04		
rs189355508	A	G	Vitamin b1 preparation	3.929E-03	6.933E-04	1.461E-08	1.458E-03	361141	8.889E-05		
rs189400778	T	C	Vitamin b1 preparation	4.966E-03	7.998E-04	5.357E-10	1.043E-03	361141	1.067E-04		
rs189471850	A	C	Vitamin b1 preparation	4.427E-03	7.671E-04	7.868E-09	1.184E-03	361141	9.222E-05		
rs190188065	C	A	Vitamin b1 preparation	2.590E-03	4.659E-04	2.700E-08	3.106E-03	361141	8.559E-05		
rs190672591	C	T	Vitamin b1 preparation	4.066E-03	7.370E-04	3.451E-08	1.321E-03	361141	8.427E-05		
rs190766552	T	A	Vitamin b1 preparation	3.937E-03	6.386E-04	7.086E-10	1.824E-03	361141	1.052E-04		
rs190934895	A	G	Vitamin b1 preparation	2.791E-03	4.856E-04	9.052E-09	3.067E-03	361141	9.147E-05		
rs192153010	C	A	Vitamin b1 preparation	2.002E-03	3.632E-04	3.561E-08	5.068E-03	361141	8.411E-05		
rs192608219	C	T	Vitamin b1 preparation	3.468E-03	5.680E-04	1.027E-09	2.087E-03	361141	1.032E-04		

SNP	Effect_ allele	Other_ allele	Id. exposure	Beta. exposure	se. exposure	pval. exposure	eaf. exposure	Sample Size	R^2	R^2sum	F_statistics
rs193028412	C	G	Vitamin b1 preparation	4.199E-03	6.670E-04	3.056E-10	1.540E-03	361141	1.098E-04		
rs199637020	C	T	Vitamin b1 preparation	6.327E-02	5.244E-03	1.638E-33	2.754E-05	361141	4.029E-04		
rs201439151	G	A	Vitamin b1 preparation	1.020E-02	1.517E-03	1.758E-11	2.883E-04	361141	1.252E-04		
rs201829379	T	C	Vitamin b1 preparation	1.353E-02	2.455E-03	3.519E-08	1.030E-04	361141	8.417E-05		
rs2230200	A	G	Vitamin b1 preparation	1.180E-02	1.639E-03	6.107E-13	2.797E-04	361141	1.435E-04		
rs35151677	A	G	Vitamin b1 preparation	7.034E-03	1.267E-03	2.857E-08	3.724E-04	361141	8.529E-05		
rs3737838	A	G	Vitamin b1 preparation	1.399E-02	2.506E-03	2.356E-08	1.300E-04	361141	8.632E-05		
rs3803964	A	G	Vitamin b1 preparation	8.675E-03	1.401E-03	5.992E-10	3.046E-04	361141	1.061E-04		
rs45486893	T	C	Vitamin b1 preparation	2.032E-02	2.999E-03	1.243E-11	6.674E-05	361141	1.271E-04		
rs4861838	A	G	Vitamin b1 preparation	4.199E-03	6.432E-04	6.667E-11	1.660E-03	361141	1.180E-04		
rs530484437	G	A	Vitamin b1 preparation	3.351E-03	5.634E-04	2.730E-09	2.141E-03	361141	9.793E-05		
rs530519995	T	C	Vitamin b1 preparation	2.167E-03	3.886E-04	2.465E-08	4.625E-03	361141	8.608E-05		
rs531232056	C	T	Vitamin b1 preparation	3.523E-03	5.537E-04	1.979E-10	2.286E-03	361141	1.121E-04		
rs531768665	A	G	Vitamin b1 preparation	6.371E-03	7.036E-04	1.384E-19	1.313E-03	361141	2.269E-04		
rs532281354	A	G	Vitamin b1 preparation	4.300E-03	6.893E-04	4.439E-10	1.438E-03	361141	1.077E-04		
rs532753641	A	G	Vitamin b1 preparation	3.874E-03	6.936E-04	2.321E-08	1.465E-03	361141	8.640E-05		
rs533669272	A	T	Vitamin b1 preparation	2.123E-03	3.866E-04	3.992E-08	4.194E-03	361141	8.349E-05		
rs539097092	A	G	Vitamin b1 preparation	3.194E-03	4.743E-04	1.642E-11	2.822E-03	361141	1.256E-04		
rs540009942	G	A	Vitamin b1 preparation	3.994E-03	7.256E-04	3.700E-08	1.319E-03	361141	8.390E-05		
rs540074743	A	G	Vitamin b1 preparation	3.780E-03	6.788E-04	2.575E-08	1.395E-03	361141	8.585E-05		
rs540443614	A	T	Vitamin b1 preparation	3.748E-03	6.763E-04	2.998E-08	1.437E-03	361141	8.503E-05		
rs545237572	C	T	Vitamin b1 preparation	2.793E-02	3.504E-03	1.580E-15	5.105E-05	361141	1.759E-04		
rs545435427	G	A	Vitamin b1 preparation	3.939E-03	6.489E-04	1.275E-09	1.756E-03	361141	1.020E-04		
rs546533901	G	T	Vitamin b1 preparation	3.239E-03	5.748E-04	1.757E-08	2.076E-03	361141	8.790E-05		
rs546637489	T	C	Vitamin b1 preparation	4.171E-03	7.241E-04	8.389E-09	1.186E-03	361141	9.188E-05		
rs547034120	G	A	Vitamin b1 preparation	3.592E-03	6.140E-04	4.895E-09	1.795E-03	361141	9.478E-05		
rs549512675	C	A	Vitamin b1 preparation	4.858E-03	6.965E-04	3.070E-12	1.425E-03	361141	1.347E-04		
rs549790609	C	A	Vitamin b1 preparation	2.622E-03	4.583E-04	1.063E-08	3.382E-03	361141	9.061E-05		
rs550246123	T	C	Vitamin b1 preparation	3.097E-03	5.051E-04	8.702E-10	2.471E-03	361141	1.041E-04		
rs555402921	G	A	Vitamin b1 preparation	3.146E-03	5.745E-04	4.366E-08	2.073E-03	361141	8.301E-05		
rs556664720	G	C	Vitamin b1 preparation	2.935E-03	4.785E-04	8.585E-10	3.155E-03	361141	1.042E-04		
rs556887177	C	T	Vitamin b1 preparation	3.336E-03	6.102E-04	4.569E-08	1.871E-03	361141	8.277E-05		
rs557781173	C	T	Vitamin b1 preparation	4.312E-03	6.495E-04	3.158E-11	1.698E-03	361141	1.220E-04		
rs560344882	C	T	Vitamin b1 preparation	2.582E-03	3.873E-04	2.607E-11	4.329E-03	361141	1.231E-04		
rs562912812	T	C	Vitamin b1 preparation	4.379E-03	7.234E-04	1.418E-09	1.302E-03	361141	1.015E-04		

SNP	Effect_ allele	Other_ allele	Id. exposure	Beta. exposure	se. exposure	pval. exposure	eaf. exposure	Sample Size	R^2	R^2sum	F_statistics
rs563915624	A	G	Vitamin b1 preparation	5.228E-03	7.838E-04	2.560E-11	1.054E-03	361141	1.232E-04		
rs565927894	G	A	Vitamin b1 preparation	3.902E-03	6.939E-04	1.872E-08	1.496E-03	361141	8.756E-05		
rs565965600	T	C	Vitamin b1 preparation	3.672E-03	6.204E-04	3.248E-09	1.703E-03	361141	9.699E-05		
rs566871822	T	C	Vitamin b1 preparation	4.605E-03	6.673E-04	5.178E-12	1.640E-03	361141	1.318E-04		
rs567764204	G	A	Vitamin b1 preparation	1.914E-03	3.417E-04	2.125E-08	5.861E-03	361141	8.688E-05		
rs568909661	T	A	Vitamin b1 preparation	2.599E-03	4.723E-04	3.716E-08	2.867E-03	361141	8.388E-05		
rs570987561	C	A	Vitamin b1 preparation	4.227E-03	6.392E-04	3.747E-11	1.793E-03	361141	1.211E-04		
rs572515321	G	T	Vitamin b1 preparation	3.922E-03	5.631E-04	3.283E-12	2.211E-03	361141	1.343E-04		
rs573265054	T	A	Vitamin b1 preparation	4.282E-03	6.912E-04	5.831E-10	1.507E-03	361141	1.063E-04		
rs574149627	G	C	Vitamin b1 preparation	3.270E-03	5.862E-04	2.437E-08	1.902E-03	361141	8.614E-05		
rs574332370	G	C	Vitamin b1 preparation	2.598E-03	4.688E-04	2.995E-08	3.083E-03	361141	8.504E-05		
rs575434257	A	G	Vitamin b1 preparation	4.636E-03	7.478E-04	5.679E-10	1.205E-03	361141	1.064E-04		
rs577798158	A	G	Vitamin b1 preparation	2.475E-03	4.538E-04	4.897E-08	3.461E-03	361141	8.239E-05		
rs62192320	C	T	Vitamin b1 preparation	3.743E-03	6.737E-04	2.764E-08	1.428E-03	361141	8.547E-05		
rs62502693	T	C	Vitamin b1 preparation	2.751E-03	4.902E-04	2.002E-08	2.594E-03	361141	8.720E-05		
rs71392173	C	T	Vitamin b1 preparation	1.722E-03	2.832E-04	1.213E-09	7.904E-03	361141	1.023E-04		
rs73276890	A	G	Vitamin b1 preparation	3.392E-03	5.966E-04	1.305E-08	1.773E-03	361141	8.950E-05		
rs73444318	C	T	Vitamin b1 preparation	3.626E-03	6.622E-04	4.348E-08	1.454E-03	361141	8.303E-05		
rs74717146	T	C	Vitamin b1 preparation	1.067E-03	1.948E-04	4.301E-08	1.613E-02	361141	8.309E-05		
rs75174177	A	G	Vitamin b1 preparation	4.348E-03	7.304E-04	2.626E-09	1.212E-03	361141	9.814E-05		
rs75228083	T	C	Vitamin b1 preparation	4.261E-03	7.368E-04	7.353E-09	1.373E-03	361141	9.259E-05		
rs75534542	A	G	Vitamin b1 preparation	2.139E-03	3.793E-04	1.716E-08	4.474E-03	361141	8.803E-05		
rs75593459	C	T	Vitamin b1 preparation	2.648E-03	4.783E-04	3.106E-08	2.896E-03	361141	8.484E-05		
rs75967563	G	A	Vitamin b1 preparation	3.353E-03	5.767E-04	6.135E-09	2.155E-03	361141	9.356E-05		
rs77686391	C	T	Vitamin b1 preparation	3.636E-03	6.365E-04	1.117E-08	1.751E-03	361141	9.034E-05		
rs77814348	T	C	Vitamin b1 preparation	7.107E-04	1.139E-04	4.374E-10	4.982E-02	361141	1.078E-04		
rs7794937	T	C	Vitamin b1 preparation	4.158E-03	7.094E-04	4.592E-09	1.326E-03	361141	9.512E-05		
rs78344684	A	G	Vitamin b1 preparation	3.430E-03	6.064E-04	1.552E-08	2.149E-03	361141	8.857E-05		
rs78610182	C	T	Vitamin b1 preparation	2.830E-03	4.426E-04	1.632E-10	3.487E-03	361141	1.131E-04		
rs79096614	G	T	Vitamin b1 preparation	2.424E-03	3.991E-04	1.245E-09	4.126E-03	361141	1.022E-04		
rs79119641	G	C	Vitamin b1 preparation	2.062E-03	3.696E-04	2.439E-08	4.886E-03	361141	8.614E-05		
rs10281751	A	G	Creon e/c granules in capsule	1.404E-03	2.254E-04	4.724E-10	1.704E-02	361141	1.074E-04	1.158E-02	37.769
rs111257239	A	T	Creon e/c granules in capsule	1.129E-03	2.056E-04	3.989E-08	1.896E-02	361141	8.349E-05		

SNP	Effect_ allele	Other_ allele	Id. exposure	Beta. exposure	se. exposure	pval. exposure	eaf. exposure	Sample Size	R^2	R^2sum	F_statistics
rs111375343	A	T	Creon e/c granules in capsule	3.970E-03	6.326E-04	3.489E-10	2.162E-03	361141	1.090E-04		
rs112505832	A	G	Creon e/c granules in capsule	8.947E-04	1.564E-04	1.072E-08	3.386E-02	361141	9.056E-05		
rs114999548	T	C	Creon e/c granules in capsule	3.495E-03	6.230E-04	2.026E-08	2.411E-03	361141	8.714E-05		
rs115694251	A	G	Creon e/c granules in capsule	4.807E-03	8.682E-04	3.085E-08	1.243E-03	361141	8.488E-05		
rs115960433	G	A	Creon e/c granules in capsule	3.034E-02	4.172E-03	3.534E-13	6.848E-05	361141	1.464E-04		
rs117240788	G	T	Creon e/c granules in capsule	4.447E-03	7.501E-04	3.048E-09	1.627E-03	361141	9.733E-05		
rs117807568	A	G	Creon e/c granules in capsule	5.084E-03	9.029E-04	1.796E-08	1.027E-03	361141	8.778E-05		
rs12142026	T	C	Creon e/c granules in capsule	1.146E-03	2.087E-04	4.034E-08	2.074E-02	361141	8.343E-05		
rs138428553	G	A	Creon e/c granules in capsule	3.354E-03	6.080E-04	3.443E-08	2.442E-03	361141	8.429E-05		
rs139911166	A	G	Creon e/c granules in capsule	3.170E-03	5.614E-04	1.637E-08	2.848E-03	361141	8.828E-05		
rs140301479	G	T	Creon e/c granules in capsule	1.028E-02	1.753E-03	4.517E-09	2.635E-04	361141	9.521E-05		
rs140739210	T	C	Creon e/c granules in capsule	3.229E-03	4.513E-04	8.423E-13	4.376E-03	361141	1.417E-04		
rs140858058	C	T	Creon e/c granules in capsule	4.400E-03	7.678E-04	1.004E-08	1.586E-03	361141	9.091E-05		
rs140900825	G	C	Creon e/c granules in capsule	3.865E-03	5.947E-04	8.140E-11	2.514E-03	361141	1.169E-04		
rs141284028	C	G	Creon e/c granules in capsule	7.245E-03	1.253E-03	7.314E-09	6.069E-04	361141	9.262E-05		
rs141695678	T	C	Creon e/c granules in capsule	2.989E-03	5.406E-04	3.226E-08	3.114E-03	361141	8.464E-05		
rs142102523	T	C	Creon e/c granules in capsule	2.736E-03	4.943E-04	3.104E-08	3.477E-03	361141	8.484E-05		
rs142305755	A	G	Creon e/c granules in capsule	3.087E-03	5.134E-04	1.814E-09	3.166E-03	361141	1.001E-04		
rs142837616	C	T	Creon e/c granules in capsule	5.283E-03	9.156E-04	7.952E-09	1.134E-03	361141	9.217E-05		
rs143748544	T	C	Creon e/c granules in capsule	5.100E-03	8.073E-04	2.653E-10	1.357E-03	361141	1.105E-04		
rs144407442	G	C	Creon e/c granules in capsule	3.074E-03	5.448E-04	1.667E-08	2.838E-03	361141	8.818E-05		
rs145875915	G	A	Creon e/c granules in capsule	4.958E-03	7.913E-04	3.713E-10	1.367E-03	361141	1.087E-04		
rs146045027	A	G	Creon e/c granules in capsule	4.125E-03	6.288E-04	5.376E-11	2.241E-03	361141	1.192E-04		

SNP	Effect_allele	Other_allele	Id. exposure	Beta. exposure	se. exposure	pval. exposure	eaf. exposure	Sample Size	R^2	R^2sum	F_statistics
rs146525983	C	G	Creon e/c granules in capsule	3.214E-03	5.703E-04	1.743E-08	2.762E-03	361141	8.794E-05		
rs146767346	G	A	Creon e/c granules in capsule	2.074E-03	3.387E-04	9.070E-10	6.920E-03	361141	1.039E-04		
rs147060044	G	A	Creon e/c granules in capsule	1.302E-03	2.240E-04	6.168E-09	1.683E-02	361141	9.354E-05		
rs147511133	T	C	Creon e/c granules in capsule	4.868E-03	7.299E-04	2.570E-11	1.730E-03	361141	1.232E-04		
rs148315214	A	G	Creon e/c granules in capsule	3.225E-03	5.588E-04	7.854E-09	3.105E-03	361141	9.223E-05		
rs148500952	C	G	Creon e/c granules in capsule	1.105E-02	1.518E-03	3.351E-13	3.920E-04	361141	1.467E-04		
rs148751644	A	G	Creon e/c granules in capsule	6.099E-03	9.718E-04	3.466E-10	8.293E-04	361141	1.091E-04		
rs149213999	T	C	Creon e/c granules in capsule	4.430E-03	7.649E-04	6.952E-09	1.531E-03	361141	9.289E-05		
rs149233111	G	T	Creon e/c granules in capsule	3.845E-03	5.338E-04	5.915E-13	2.998E-03	361141	1.436E-04		
rs149252567	C	T	Creon e/c granules in capsule	3.061E-03	5.165E-04	3.099E-09	3.313E-03	361141	9.724E-05		
rs182105960	A	G	Creon e/c granules in capsule	6.398E-03	9.006E-04	1.218E-12	1.183E-03	361141	1.397E-04		
rs183192493	C	T	Creon e/c granules in capsule	2.282E-03	3.748E-04	1.139E-09	6.454E-03	361141	1.026E-04		
rs183378252	G	C	Creon e/c granules in capsule	3.868E-03	6.495E-04	2.587E-09	1.927E-03	361141	9.822E-05		
rs183637928	C	T	Creon e/c granules in capsule	3.503E-03	6.243E-04	2.009E-08	2.268E-03	361141	8.718E-05		
rs183698494	T	C	Creon e/c granules in capsule	2.848E-03	5.014E-04	1.354E-08	3.601E-03	361141	8.930E-05		
rs184098030	T	A	Creon e/c granules in capsule	3.096E-03	5.209E-04	2.787E-09	3.221E-03	361141	9.782E-05		
rs184116715	T	C	Creon e/c granules in capsule	5.485E-03	8.443E-04	8.269E-11	1.214E-03	361141	1.168E-04		
rs184577859	T	C	Creon e/c granules in capsule	4.782E-03	8.708E-04	3.990E-08	1.278E-03	361141	8.349E-05		
rs184629391	T	C	Creon e/c granules in capsule	4.025E-03	7.378E-04	4.882E-08	1.622E-03	361141	8.241E-05		
rs184695554	C	G	Creon e/c granules in capsule	4.219E-03	7.622E-04	3.106E-08	1.480E-03	361141	8.484E-05		
rs184697038	T	G	Creon e/c granules in capsule	3.750E-03	6.038E-04	5.270E-10	2.486E-03	361141	1.068E-04		
rs184854377	C	T	Creon e/c granules in capsule	3.469E-03	6.092E-04	1.235E-08	2.478E-03	361141	8.980E-05		
rs185775208	G	C	Creon e/c granules in capsule	3.175E-03	5.269E-04	1.685E-09	3.147E-03	361141	1.005E-04		

SNP	Effect_ allele	Other_ allele	Id. exposure	Beta. exposure	se. exposure	pval. exposure	eaf. exposure	Sample Size	R^2	R^2sum	*F*_statistics
rs185845022	T	C	Creon e/c granules in capsule	4.317E-03	7.625E-04	1.501E-08	1.661E-03	361141	8.875E-05		
rs186859265	A	C	Creon e/c granules in capsule	4.894E-03	8.179E-04	2.177E-09	1.298E-03	361141	9.915E-05		
rs187027935	A	G	Creon e/c granules in capsule	5.569E-03	7.777E-04	8.015E-13	1.511E-03	361141	1.420E-04		
rs187037180	A	G	Creon e/c granules in capsule	4.720E-03	7.094E-04	2.869E-11	1.766E-03	361141	1.226E-04		
rs187358164	C	G	Creon e/c granules in capsule	3.504E-03	6.308E-04	2.787E-08	2.265E-03	361141	8.542E-05		
rs188004632	T	C	Creon e/c granules in capsule	2.768E-03	4.978E-04	2.703E-08	3.786E-03	361141	8.559E-05		
rs188668943	A	G	Creon e/c granules in capsule	5.222E-03	7.960E-04	5.362E-11	1.424E-03	361141	1.192E-04		
rs188806541	A	G	Creon e/c granules in capsule	6.957E-03	9.517E-04	2.671E-13	1.021E-03	361141	1.480E-04		
rs189382075	T	A	Creon e/c granules in capsule	4.794E-03	8.061E-04	2.718E-09	1.451E-03	361141	9.795E-05		
rs189542231	A	C	Creon e/c granules in capsule	3.302E-03	5.624E-04	4.318E-09	2.656E-03	361141	9.546E-05		
rs189647524	T	G	Creon e/c granules in capsule	3.450E-03	6.289E-04	4.138E-08	2.181E-03	361141	8.330E-05		
rs189924416	A	G	Creon e/c granules in capsule	4.842E-03	8.838E-04	4.297E-08	1.082E-03	361141	8.310E-05		
rs190402679	G	A	Creon e/c granules in capsule	2.123E-03	3.571E-04	2.761E-09	6.370E-03	361141	9.787E-05		
rs191023875	T	C	Creon e/c granules in capsule	2.865E-03	5.034E-04	1.262E-08	3.298E-03	361141	8.968E-05		
rs191361633	T	C	Creon e/c granules in capsule	3.476E-03	4.376E-04	1.975E-15	4.253E-03	361141	1.747E-04		
rs191878277	T	C	Creon e/c granules in capsule	2.748E-03	5.038E-04	4.923E-08	3.391E-03	361141	8.237E-05		
rs192102398	G	A	Creon e/c granules in capsule	3.498E-03	6.319E-04	3.101E-08	2.276E-03	361141	8.485E-05		
rs192406923	C	T	Creon e/c granules in capsule	4.373E-03	5.971E-04	2.420E-13	2.483E-03	361141	1.485E-04		
rs193007666	T	G	Creon e/c granules in capsule	4.653E-03	7.724E-04	1.702E-09	1.505E-03	361141	1.005E-04		
rs199551268	A	T	Creon e/c granules in capsule	3.156E-02	5.186E-03	1.156E-09	3.647E-05	361141	1.026E-04		
rs199754207	A	G	Creon e/c granules in capsule	9.183E-03	1.672E-03	3.956E-08	3.000E-04	361141	8.354E-05		
rs2035846	G	A	Creon e/c granules in capsule	5.357E-03	8.807E-04	1.180E-09	1.387E-03	361141	1.025E-04		
rs35833234	A	G	Creon e/c granules in capsule	1.989E-03	3.633E-04	4.410E-08	6.429E-03	361141	8.296E-05		

SNP	Effect_ allele	Other_ allele	Id. exposure	Beta. exposure	se. exposure	pval. exposure	eaf. exposure	Sample Size	R^2	R^2sum	*F*_statistics
rs36124334	T	C	Creon e/c granules in capsule	3.022E-03	4.915E-04	7.809E-10	3.743E-03	361141	1.047E-04		
rs369522081	C	T	Creon e/c granules in capsule	4.316E-03	7.846E-04	3.785E-08	1.513E-03	361141	8.378E-05		
rs372855754	C	G	Creon e/c granules in capsule	2.224E-03	3.935E-04	1.593E-08	5.494E-03	361141	8.843E-05		
rs3738178	A	T	Creon e/c granules in capsule	1.374E-02	2.024E-03	1.122E-11	2.368E-04	361141	1.277E-04		
rs528304267	C	A	Creon e/c granules in capsule	3.303E-03	5.293E-04	4.372E-10	3.078E-03	361141	1.078E-04		
rs529271432	C	G	Creon e/c granules in capsule	6.277E-03	9.055E-04	4.163E-12	1.146E-03	361141	1.330E-04		
rs531195540	C	T	Creon e/c granules in capsule	5.276E-03	9.127E-04	7.473E-09	1.026E-03	361141	9.250E-05		
rs531351150	A	G	Creon e/c granules in capsule	3.518E-03	6.378E-04	3.473E-08	2.017E-03	361141	8.424E-05		
rs532180310	G	A	Creon e/c granules in capsule	2.821E-03	4.836E-04	5.475E-09	3.762E-03	361141	9.418E-05		
rs534148559	A	C	Creon e/c granules in capsule	4.005E-03	7.263E-04	3.515E-08	1.669E-03	361141	8.418E-05		
rs535145896	T	C	Creon e/c granules in capsule	3.489E-03	4.997E-04	2.906E-12	3.446E-03	361141	1.350E-04		
rs536195307	A	G	Creon e/c granules in capsule	4.955E-03	8.037E-04	7.059E-10	1.286E-03	361141	1.052E-04		
rs540446471	A	G	Creon e/c granules in capsule	4.640E-03	7.181E-04	1.038E-10	1.658E-03	361141	1.156E-04		
rs542672985	G	A	Creon e/c granules in capsule	4.708E-03	7.856E-04	2.064E-09	1.526E-03	361141	9.944E-05		
rs543583350	T	C	Creon e/c granules in capsule	4.541E-03	8.065E-04	1.797E-08	1.381E-03	361141	8.778E-05		
rs546037422	G	A	Creon e/c granules in capsule	7.032E-03	9.432E-04	9.011E-14	1.089E-03	361141	1.539E-04		
rs547073731	T	C	Creon e/c granules in capsule	7.161E-03	8.588E-04	7.527E-17	1.239E-03	361141	1.925E-04		
rs551101776	C	A	Creon e/c granules in capsule	5.112E-03	9.138E-04	2.209E-08	1.143E-03	361141	8.667E-05		
rs553185095	G	A	Creon e/c granules in capsule	3.892E-02	4.722E-03	1.688E-16	3.797E-05	361141	1.881E-04		
rs553298491	T	C	Creon e/c granules in capsule	2.548E-03	4.624E-04	3.580E-08	3.938E-03	361141	8.408E-05		
rs553597885	T	C	Creon e/c granules in capsule	4.171E-03	6.843E-04	1.088E-09	2.004E-03	361141	1.029E-04		
rs560090417	A	G	Creon e/c granules in capsule	5.242E-03	8.205E-04	1.677E-10	1.312E-03	361141	1.130E-04		
rs560838364	C	G	Creon e/c granules in capsule	5.115E-03	8.022E-04	1.815E-10	1.332E-03	361141	1.126E-04		

SNP	Effect_ allele	Other_ allele	Id. exposure	Beta. exposure	se. exposure	pval. exposure	eaf. exposure	Sample Size	R^2	R^2sum	F_statistics
rs563051284	T	C	Creon e/c granules in capsule	3.550E-03	5.606E-04	2.402E-10	2.928E-03	361141	1.111E-04		
rs567781656	A	G	Creon e/c granules in capsule	2.898E-03	5.251E-04	3.431E-08	3.227E-03	361141	8.430E-05		
rs568255087	G	A	Creon e/c granules in capsule	4.191E-03	7.522E-04	2.525E-08	1.451E-03	361141	8.595E-05		
rs569947109	C	T	Creon e/c granules in capsule	5.545E-03	8.524E-04	7.753E-11	1.252E-03	361141	1.172E-04		
rs570537792	A	G	Creon e/c granules in capsule	4.258E-03	7.670E-04	2.831E-08	1.583E-03	361141	8.534E-05		
rs574476721	T	C	Creon e/c granules in capsule	5.127E-03	8.952E-04	1.023E-08	1.136E-03	361141	9.081E-05		
rs575540373	A	G	Creon e/c granules in capsule	5.557E-03	8.325E-04	2.476E-11	1.290E-03	361141	1.234E-04		
rs577524886	G	T	Creon e/c granules in capsule	3.987E-03	6.759E-04	3.671E-09	1.896E-03	361141	9.633E-05		
rs58163905	G	T	Creon e/c granules in capsule	5.227E-03	9.056E-04	7.826E-09	1.070E-03	361141	9.225E-05		
rs606815	A	A	Creon e/c granules in capsule	-3.369E-04	5.881E-05	1.015E-08	3.501E-01	361141	9.086E-05		
rs62184084	A	G	Creon e/c granules in capsule	3.427E-03	6.220E-04	3.576E-08	2.242E-03	361141	8.408E-05		
rs72681723	G	A	Creon e/c granules in capsule	2.043E-03	3.674E-04	2.702E-08	6.281E-03	361141	8.559E-05		
rs73112523	T	G	Creon e/c granules in capsule	5.190E-03	7.869E-04	4.249E-11	1.524E-03	361141	1.204E-04		
rs75469927	C	T	Creon e/c granules in capsule	2.026E-03	3.148E-04	1.242E-10	8.060E-03	361141	1.146E-04		
rs77503961	A	G	Creon e/c granules in capsule	5.005E-03	8.946E-04	2.217E-08	1.223E-03	361141	8.665E-05		
rs78074574	G	C	Creon e/c granules in capsule	2.449E-03	4.432E-04	3.299E-08	4.088E-03	361141	8.451E-05		
rs79922863	T	G	Creon e/c granules in capsule	3.583E-03	5.781E-04	5.717E-10	2.852E-03	361141	1.064E-04		
rs80166300	G	C	Creon e/c granules in capsule	3.442E-03	5.776E-04	2.542E-09	2.649E-03	361141	9.831E-05		
rs111965614	G	T	Pentasa sr 250mg m/r tablet	2.319E-03	4.155E-04	2.378E-08	5.552E-03	361141	8.628E-05	1.037E-02	38.621
rs114073919	T	G	Pentasa sr 250mg m/r tablet	5.705E-03	1.008E-03	1.499E-08	1.031E-03	361141	8.876E-05		
rs115544261	C	G	Pentasa sr 250mg m/r tablet	3.561E-03	6.063E-04	4.265E-09	2.755E-03	361141	9.552E-05		
rs116972960	T	C	Pentasa sr 250mg m/r tablet	3.491E-03	5.002E-04	2.960E-12	4.091E-03	361141	1.349E-04		
rs117226873	A	T	Pentasa sr 250mg m/r tablet	5.180E-03	8.513E-04	1.166E-09	1.579E-03	361141	1.025E-04		

SNP	Effect_ allele	Other_ allele	Id. exposure	Beta. exposure	se. exposure	pval. exposure	eaf. exposure	Sample Size	R^2	R^2sum	F_statistics
rs117410356	T	C	Pentasa sr 250mg m/r tablet	1.224E-02	2.109E-03	6.501E-09	2.146E-04	361141	9.325E-05		
rs117783846	T	C	Pentasa sr 250mg m/r tablet	4.093E-03	7.148E-04	1.026E-08	2.144E-03	361141	9.080E-05		
rs12263552	G	A	Pentasa sr 250mg m/r tablet	4.986E-03	7.513E-04	3.227E-11	1.775E-03	361141	1.219E-04		
rs137880301	A	C	Pentasa sr 250mg m/r tablet	4.170E-03	7.623E-04	4.503E-08	1.839E-03	361141	8.284E-05		
rs138391107	G	A	Pentasa sr 250mg m/r tablet	3.590E-03	6.574E-04	4.759E-08	2.306E-03	361141	8.255E-05		
rs138462199	C	G	Pentasa sr 250mg m/r tablet	2.825E-03	5.001E-04	1.612E-08	4.500E-03	361141	8.836E-05		
rs138754417	T	C	Pentasa sr 250mg m/r tablet	2.623E-03	4.552E-04	8.319E-09	4.617E-03	361141	9.192E-05		
rs139148832	G	A	Pentasa sr 250mg m/r tablet	2.405E-03	4.309E-04	2.406E-08	5.963E-03	361141	8.621E-05		
rs139245880	T	C	Pentasa sr 250mg m/r tablet	1.617E-03	2.882E-04	1.998E-08	1.404E-02	361141	8.721E-05		
rs140358676	T	C	Pentasa sr 250mg m/r tablet	3.991E-03	7.078E-04	1.722E-08	2.081E-03	361141	8.801E-05		
rs141563618	T	C	Pentasa sr 250mg m/r tablet	1.794E-02	2.543E-03	1.741E-12	1.789E-04	361141	1.378E-04		
rs141641146	G	T	Pentasa sr 250mg m/r tablet	2.569E-03	4.604E-04	2.405E-08	4.896E-03	361141	8.621E-05		
rs142232463	C	A	Pentasa sr 250mg m/r tablet	5.716E-03	9.487E-04	1.691E-09	1.235E-03	361141	1.005E-04		
rs142645322	A	C	Pentasa sr 250mg m/r tablet	5.898E-03	9.671E-04	1.066E-09	1.203E-03	361141	1.030E-04		
rs142933925	C	T	Pentasa sr 250mg m/r tablet	6.234E-03	1.001E-03	4.813E-10	1.127E-03	361141	1.073E-04		
rs143083095	G	A	Pentasa sr 250mg m/r tablet	4.525E-03	8.083E-04	2.178E-08	1.742E-03	361141	8.675E-05		
rs143621313	G	T	Pentasa sr 250mg m/r tablet	5.712E-03	9.540E-04	2.132E-09	1.184E-03	361141	9.926E-05		
rs145313529	T	G	Pentasa sr 250mg m/r tablet	5.767E-03	9.586E-04	1.793E-09	1.103E-03	361141	1.002E-04		
rs145558250	G	C	Pentasa sr 250mg m/r tablet	3.740E-03	6.777E-04	3.412E-08	2.234E-03	361141	8.434E-05		
rs145623213	G	A	Pentasa sr 250mg m/r tablet	2.314E-02	4.051E-03	1.115E-08	5.815E-05	361141	9.035E-05		
rs145696562	G	A	Pentasa sr 250mg m/r tablet	2.111E-03	3.434E-04	7.847E-10	9.309E-03	361141	1.047E-04		
rs145798628	C	T	Pentasa sr 250mg m/r tablet	4.457E-03	6.837E-04	7.105E-11	2.169E-03	361141	1.176E-04		
rs145967390	T	A	Pentasa sr 250mg m/r tablet	1.207E-02	2.103E-03	9.502E-09	2.332E-04	361141	9.121E-05		

SNP	Effect_ allele	Other_ allele	Id. exposure	Beta. exposure	se. exposure	pval. exposure	eaf. exposure	Sample Size	R^2	R^2sum	*F*_statistics
rs146095268	C	T	Pentasa sr 250mg m/r tablet	4.630E-03	7.561E-04	9.142E-10	2.057E-03	361141	1.038E-04		
rs147100213	G	C	Pentasa sr 250mg m/r tablet	4.520E-03	5.853E-04	1.144E-14	3.169E-03	361141	1.651E-04		
rs147613723	G	C	Pentasa sr 250mg m/r tablet	3.781E-02	5.157E-03	2.262E-13	3.828E-05	361141	1.489E-04		
rs147667071	C	A	Pentasa sr 250mg m/r tablet	6.296E-03	1.042E-03	1.538E-09	1.015E-03	361141	1.010E-04		
rs147716724	A	G	Pentasa sr 250mg m/r tablet	2.253E-02	4.010E-03	1.909E-08	6.476E-05	361141	8.746E-05		
rs148235754	G	A	Pentasa sr 250mg m/r tablet	5.434E-03	9.456E-04	9.122E-09	1.248E-03	361141	9.143E-05		
rs148298809	T	C	Pentasa sr 250mg m/r tablet	6.433E-03	1.128E-03	1.160E-08	8.319E-04	361141	9.013E-05		
rs150271689	A	G	Pentasa sr 250mg m/r tablet	6.022E-03	1.022E-03	3.765E-09	1.117E-03	361141	9.619E-05		
rs17116167	A	C	Pentasa sr 250mg m/r tablet	4.328E-03	7.209E-04	1.927E-09	2.099E-03	361141	9.981E-05		
rs181402654	T	C	Pentasa sr 250mg m/r tablet	3.301E-03	5.855E-04	1.716E-08	3.372E-03	361141	8.803E-05		
rs181591879	G	C	Pentasa sr 250mg m/r tablet	2.080E-02	2.722E-03	2.171E-14	1.288E-04	361141	1.616E-04		
rs181617731	A	C	Pentasa sr 250mg m/r tablet	6.028E-03	9.630E-04	3.868E-10	1.177E-03	361141	1.085E-04		
rs182447742	A	T	Pentasa sr 250mg m/r tablet	5.179E-03	9.299E-04	2.556E-08	1.198E-03	361141	8.589E-05		
rs183653380	T	C	Pentasa sr 250mg m/r tablet	4.431E-03	7.524E-04	3.889E-09	1.933E-03	361141	9.602E-05		
rs183799965	A	G	Pentasa sr 250mg m/r tablet	5.233E-03	8.363E-04	3.919E-10	1.608E-03	361141	1.084E-04		
rs184590639	A	C	Pentasa sr 250mg m/r tablet	4.090E-03	7.424E-04	3.606E-08	2.036E-03	361141	8.404E-05		
rs184630148	T	C	Pentasa sr 250mg m/r tablet	3.226E-03	5.896E-04	4.450E-08	3.381E-03	361141	8.291E-05		
rs185659258	A	G	Pentasa sr 250mg m/r tablet	3.861E-03	6.711E-04	8.767E-09	2.183E-03	361141	9.164E-05		
rs185811507	C	T	Pentasa sr 250mg m/r tablet	5.391E-03	7.654E-04	1.877E-12	1.749E-03	361141	1.374E-04		
rs185889336	G	A	Pentasa sr 250mg m/r tablet	2.537E-03	4.141E-04	8.980E-10	6.665E-03	361141	1.039E-04		
rs186181126	T	C	Pentasa sr 250mg m/r tablet	5.061E-03	8.823E-04	9.698E-09	1.425E-03	361141	9.110E-05		
rs186607333	C	G	Pentasa sr 250mg m/r tablet	3.405E-03	6.058E-04	1.915E-08	2.965E-03	361141	8.744E-05		
rs186969775	A	G	Pentasa sr 250mg m/r tablet	3.045E-03	4.526E-04	1.731E-11	5.789E-03	361141	1.253E-04		

SNP	Effect_ allele	Other_ allele	Id. exposure	Beta. exposure	se. exposure	pval. exposure	eaf. exposure	Sample Size	R^2	R^2sum	F_statistics
rs187820773	C	A	Pentasa sr 250mg m/r tablet	3.724E-03	6.281E-04	3.057E-09	2.741E-03	361141	9.732E-05		
rs187922924	G	A	Pentasa sr 250mg m/r tablet	3.587E-03	4.601E-04	6.384E-15	5.119E-03	361141	1.683E-04		
rs188364229	A	G	Pentasa sr 250mg m/r tablet	3.581E-03	6.209E-04	8.070E-09	2.796E-03	361141	9.209E-05		
rs188546925	T	C	Pentasa sr 250mg m/r tablet	2.126E-03	3.582E-04	2.922E-09	8.093E-03	361141	9.756E-05		
rs189104238	C	T	Pentasa sr 250mg m/r tablet	5.554E-03	1.009E-03	3.697E-08	1.155E-03	361141	8.390E-05		
rs191719596	T	C	Pentasa sr 250mg m/r tablet	6.439E-03	9.246E-04	3.313E-12	1.366E-03	361141	1.343E-04		
rs191738384	C	T	Pentasa sr 250mg m/r tablet	4.349E-03	7.613E-04	1.110E-08	1.877E-03	361141	9.037E-05		
rs192161920	G	A	Pentasa sr 250mg m/r tablet	5.920E-03	8.986E-04	4.473E-11	1.386E-03	361141	1.202E-04		
rs192573293	T	C	Pentasa sr 250mg m/r tablet	5.127E-03	9.135E-04	1.988E-08	1.310E-03	361141	8.724E-05		
rs200288891	T	G	Pentasa sr 250mg m/r tablet	4.908E-03	8.389E-04	4.907E-09	1.673E-03	361141	9.477E-05		
rs200678581	T	C	Pentasa sr 250mg m/r tablet	1.403E-02	2.262E-03	5.500E-10	2.124E-04	361141	1.066E-04		
rs201119350	A	G	Pentasa sr 250mg m/r tablet	1.145E-02	2.011E-03	1.238E-08	2.900E-04	361141	8.979E-05		
rs2242030	A	G	Pentasa sr 250mg m/r tablet	3.506E-02	4.961E-03	1.592E-12	3.877E-05	361141	1.382E-04		
rs28417825	T	C	Pentasa sr 250mg m/r tablet	3.875E-03	7.001E-04	3.125E-08	2.014E-03	361141	8.481E-05		
rs35733104	T	C	Pentasa sr 250mg m/r tablet	2.189E-02	2.791E-03	4.384E-15	1.374E-04	361141	1.703E-04		
rs367632712	C	T	Pentasa sr 250mg m/r tablet	3.170E-03	5.727E-04	3.131E-08	3.436E-03	361141	8.480E-05		
rs377559702	A	G	Pentasa sr 250mg m/r tablet	2.658E-02	4.372E-03	1.202E-09	8.230E-05	361141	1.024E-04		
rs527266131	G	T	Pentasa sr 250mg m/r tablet	5.181E-03	8.270E-04	3.730E-10	1.474E-03	361141	1.087E-04		
rs529083628	G	A	Pentasa sr 250mg m/r tablet	2.843E-03	4.997E-04	1.272E-08	4.173E-03	361141	8.964E-05		
rs529781387	G	C	Pentasa sr 250mg m/r tablet	5.675E-03	9.450E-04	1.908E-09	1.269E-03	361141	9.986E-05		
rs530137520	A	G	Pentasa sr 250mg m/r tablet	5.049E-03	8.893E-04	1.371E-08	1.494E-03	361141	8.924E-05		
rs543274794	C	T	Pentasa sr 250mg m/r tablet	5.110E-03	8.980E-04	1.273E-08	1.279E-03	361141	8.964E-05		
rs545237572	C	T	Pentasa sr 250mg m/r tablet	2.775E-02	4.427E-03	3.662E-10	5.105E-05	361141	1.088E-04		

SNP	Effect_allele	Other_allele	Id. exposure	Beta. exposure	se. exposure	pval. exposure	eaf. exposure	Sample Size	R^2	R^2sum	F_statistics
rs548385285	T	A	Pentasa sr 250mg m/r tablet	6.807E-03	9.576E-04	1.172E-12	1.215E-03	361141	1.399E-04		
rs549310858	T	C	Pentasa sr 250mg m/r tablet	4.363E-03	7.676E-04	1.314E-08	1.770E-03	361141	8.946E-05		
rs552773438	T	G	Pentasa sr 250mg m/r tablet	4.492E-03	8.128E-04	3.277E-08	1.646E-03	361141	8.455E-05		
rs553138419	C	T	Pentasa sr 250mg m/r tablet	5.446E-03	8.091E-04	1.693E-11	1.627E-03	361141	1.254E-04		
rs553963520	C	G	Pentasa sr 250mg m/r tablet	4.891E-03	7.467E-04	5.743E-11	1.917E-03	361141	1.188E-04		
rs555347031	C	A	Pentasa sr 250mg m/r tablet	4.346E-03	7.967E-04	4.904E-08	1.636E-03	361141	8.239E-05		
rs557736648	A	G	Pentasa sr 250mg m/r tablet	5.043E-03	8.934E-04	1.657E-08	1.277E-03	361141	8.822E-05		
rs563156776	A	G	Pentasa sr 250mg m/r tablet	5.077E-03	8.965E-04	1.494E-08	1.439E-03	361141	8.877E-05		
rs564269939	A	T	Pentasa sr 250mg m/r tablet	5.943E-03	9.616E-04	6.404E-10	1.207E-03	361141	1.058E-04		
rs571015848	G	A	Pentasa sr 250mg m/r tablet	3.942E-03	6.799E-04	6.720E-09	2.354E-03	361141	9.307E-05		
rs571203333	C	A	Pentasa sr 250mg m/r tablet	4.146E-03	7.468E-04	2.834E-08	1.939E-03	361141	8.533E-05		
rs572727593	T	C	Pentasa sr 250mg m/r tablet	3.855E-03	6.815E-04	1.542E-08	2.450E-03	361141	8.860E-05		
rs574829619	A	G	Pentasa sr 250mg m/r tablet	6.597E-03	9.479E-04	3.413E-12	1.224E-03	361141	1.341E-04		
rs576424928	C	T	Pentasa sr 250mg m/r tablet	3.873E-03	7.058E-04	4.075E-08	2.271E-03	361141	8.338E-05		
rs577151236	A	G	Pentasa sr 250mg m/r tablet	5.814E-03	8.224E-04	1.556E-12	1.668E-03	361141	1.384E-04		
rs615783	T	C	Pentasa sr 250mg m/r tablet	4.503E-03	7.502E-04	1.945E-09	1.860E-03	361141	9.976E-05		
rs6558058	T	G	Pentasa sr 250mg m/r tablet	5.626E-03	9.969E-04	1.673E-08	1.596E-03	361141	8.817E-05		
rs72627210	G	A	Pentasa sr 250mg m/r tablet	4.770E-02	5.787E-03	1.707E-16	3.483E-05	361141	1.880E-04		
rs73089628	G	A	Pentasa sr 250mg m/r tablet	5.086E-03	7.508E-04	1.262E-11	2.023E-03	361141	1.270E-04		
rs7653164	A	C	Pentasa sr 250mg m/r tablet	2.193E-02	2.281E-03	7.017E-22	2.135E-04	361141	2.559E-04		
rs77263020	G	T	Pentasa sr 250mg m/r tablet	7.110E-03	9.505E-04	7.426E-14	1.160E-03	361141	1.549E-04		
rs79140567	G	T	Pentasa sr 250mg m/r tablet	5.743E-03	9.462E-04	1.283E-09	1.117E-03	361141	1.020E-04		
rs79437925	A	T	Pentasa sr 250mg m/r tablet	2.358E-03	3.943E-04	2.224E-09	6.632E-03	361141	9.903E-05		

SNP	Effect_ allele	Other_ allele	Id. exposure	Beta. exposure	se. exposure	pval. exposure	eaf. exposure	Sample Size	R^2	R^2sum	*F*_statistics
rs9511501	C	T	Pentasa sr 250mg m/r tablet	-5.547E-03	8.480E-04	6.095E-11	1.358E-03	361141	1.185E-04		
rs113222110	T	A	Zolmitriptan	1.738E-03	3.117E-04	2.464E-08	1.495E-02	361141	8.608E-05	7.054E-03	37.176
rs113683806	T	C	Zolmitriptan	7.069E-03	1.120E-03	2.759E-10	1.227E-03	361141	1.103E-04		
rs115149116	G	A	Zolmitriptan	6.490E-03	1.138E-03	1.169E-08	1.370E-03	361141	9.009E-05		
rs117358680	C	T	Zolmitriptan	3.731E-03	6.524E-04	1.070E-08	3.344E-03	361141	9.057E-05		
rs117408069	T	A	Zolmitriptan	4.154E-03	7.610E-04	4.798E-08	2.451E-03	361141	8.250E-05		
rs139196947	T	C	Zolmitriptan	5.544E-03	1.002E-03	3.131E-08	1.428E-03	361141	8.480E-05		
rs139350836	A	G	Zolmitriptan	1.950E-03	3.508E-04	2.727E-08	1.135E-02	361141	8.554E-05		
rs139374068	T	C	Zolmitriptan	4.760E-03	8.299E-04	9.697E-09	2.074E-03	361141	9.110E-05		
rs139996419	A	G	Zolmitriptan	1.073E-02	1.927E-03	2.587E-08	3.558E-04	361141	8.582E-05		
rs140924076	C	G	Zolmitriptan	3.806E-02	6.310E-03	1.626E-09	3.874E-05	361141	1.007E-04		
rs142477441	G	T	Zolmitriptan	6.134E-03	9.358E-04	5.586E-11	1.681E-03	361141	1.190E-04		
rs144310107	T	C	Zolmitriptan	9.316E-03	1.067E-03	2.565E-18	1.416E-03	361141	2.110E-04		
rs144469974	C	G	Zolmitriptan	2.962E-03	4.908E-04	1.593E-09	6.020E-03	361141	1.008E-04		
rs145105181	A	G	Zolmitriptan	5.657E-03	1.036E-03	4.793E-08	1.385E-03	361141	8.251E-05		
rs145109511	T	C	Zolmitriptan	1.952E-03	3.502E-04	2.517E-08	1.234E-02	361141	8.597E-05		
rs145940190	C	T	Zolmitriptan	5.078E-03	9.259E-04	4.144E-08	1.788E-03	361141	8.329E-05		
rs146924732	A	G	Zolmitriptan	5.795E-03	1.034E-03	2.077E-08	1.453E-03	361141	8.700E-05		
rs148197945	G	A	Zolmitriptan	5.581E-03	8.727E-04	1.599E-10	2.039E-03	361141	1.133E-04		
rs150426437	A	G	Zolmitriptan	3.754E-03	6.762E-04	2.825E-08	3.554E-03	361141	8.535E-05		
rs16926453	C	T	Zolmitriptan	5.580E-02	7.346E-03	3.040E-14	2.730E-05	361141	1.598E-04		
rs181942142	A	G	Zolmitriptan	5.399E-03	9.590E-04	1.808E-08	1.605E-03	361141	8.775E-05		
rs182078879	T	G	Zolmitriptan	1.996E-03	3.630E-04	3.825E-08	1.150E-02	361141	8.372E-05		
rs182085092	T	C	Zolmitriptan	3.559E-03	6.259E-04	1.304E-08	3.600E-03	361141	8.951E-05		
rs182630702	G	A	Zolmitriptan	5.975E-03	9.350E-04	1.652E-10	1.634E-03	361141	1.131E-04		
rs182996356	T	C	Zolmitriptan	1.835E-02	3.028E-03	1.351E-09	1.747E-04	361141	1.017E-04		
rs183116520	T	C	Zolmitriptan	4.071E-03	7.374E-04	3.383E-08	2.732E-03	361141	8.438E-05		
rs183420538	A	C	Zolmitriptan	7.244E-03	1.087E-03	2.668E-11	1.383E-03	361141	1.230E-04		
rs185386236	A	G	Zolmitriptan	4.031E-03	7.092E-04	1.324E-08	3.023E-03	361141	8.942E-05		
rs185864561	C	T	Zolmitriptan	6.230E-03	1.071E-03	6.032E-09	1.389E-03	361141	9.366E-05		
rs187747829	G	A	Zolmitriptan	6.399E-03	1.058E-03	1.484E-09	1.418E-03	361141	1.012E-04		
rs188395338	C	T	Zolmitriptan	4.668E-03	8.539E-04	4.593E-08	2.134E-03	361141	8.274E-05		
rs189551357	A	G	Zolmitriptan	6.222E-03	1.031E-03	1.610E-09	1.457E-03	361141	1.008E-04		
rs189892332	G	A	Zolmitriptan	3.280E-03	5.410E-04	1.335E-09	5.103E-03	361141	1.018E-04		
rs190225584	C	T	Zolmitriptan	4.459E-03	6.985E-04	1.731E-10	3.317E-03	361141	1.128E-04		
rs190685084	A	C	Zolmitriptan	4.825E-03	8.540E-04	1.599E-08	2.166E-03	361141	8.841E-05		
rs191294437	T	G	Zolmitriptan	4.083E-03	7.352E-04	2.800E-08	2.604E-03	361141	8.540E-05		
rs191334623	A	G	Zolmitriptan	5.273E-03	9.185E-04	9.440E-09	1.905E-03	361141	9.124E-05		
rs191539964	T	G	Zolmitriptan	6.686E-03	1.217E-03	3.906E-08	9.953E-04	361141	8.361E-05		
rs192178946	C	G	Zolmitriptan	3.672E-03	6.268E-04	4.690E-09	3.630E-03	361141	9.501E-05		
rs192489514	C	G	Zolmitriptan	3.536E-03	6.301E-04	2.006E-08	3.471E-03	361141	8.719E-05		
rs192807512	C	T	Zolmitriptan	7.189E-03	1.112E-03	9.962E-11	1.249E-03	361141	1.158E-04		
rs192852513	A	G	Zolmitriptan	4.436E-03	7.769E-04	1.135E-08	2.501E-03	361141	9.025E-05		
rs199700360	A	G	Zolmitriptan	6.047E-03	9.791E-04	6.561E-10	1.379E-03	361141	1.056E-04		
rs201353657	T	C	Zolmitriptan	2.980E-02	5.450E-03	4.556E-08	5.300E-05	361141	8.278E-05		
rs373519408	G	T	Zolmitriptan	5.930E-03	8.826E-04	1.840E-11	1.865E-03	361141	1.250E-04		
rs45486893	T	C	Zolmitriptan	4.050E-02	4.457E-03	1.025E-19	6.674E-05	361141	2.286E-04		
rs45570942	T	C	Zolmitriptan	3.216E-02	3.250E-03	4.406E-23	1.284E-04	361141	2.710E-04		
rs534302873	T	C	Zolmitriptan	3.236E-03	5.825E-04	2.783E-08	4.686E-03	361141	8.543E-05		

SNP	Effect_ allele	Other_ allele	Id. exposure	Beta. exposure	se. exposure	pval. exposure	eaf. exposure	Sample Size	R^2	R^2sum	F_statistics
rs536312318	A	G	Zolmitriptan	4.514E-03	8.198E-04	3.667E-08	2.145E-03	361141	8.395E-05		
rs538858088	A	G	Zolmitriptan	3.081E-03	5.360E-04	9.071E-09	5.123E-03	361141	9.146E-05		
rs547361759	C	T	Zolmitriptan	5.590E-03	1.022E-03	4.517E-08	1.529E-03	361141	8.283E-05		
rs550345407	T	A	Zolmitriptan	7.187E-03	1.219E-03	3.692E-09	9.942E-04	361141	9.630E-05		
rs551217133	A	G	Zolmitriptan	6.485E-03	1.123E-03	7.768E-09	1.167E-03	361141	9.229E-05		
rs559805874	G	A	Zolmitriptan	3.808E-03	6.888E-04	3.238E-08	2.985E-03	361141	8.462E-05		
rs56113397	C	T	Zolmitriptan	3.598E-03	6.000E-04	2.013E-09	4.267E-03	361141	9.957E-05		
rs561761332	A	G	Zolmitriptan	7.468E-03	1.054E-03	1.401E-12	1.471E-03	361141	1.389E-04		
rs563896780	A	G	Zolmitriptan	5.838E-03	1.043E-03	2.170E-08	1.392E-03	361141	8.677E-05		
rs564146046	C	T	Zolmitriptan	6.911E-03	1.072E-03	1.133E-10	1.316E-03	361141	1.151E-04		
rs571847018	T	C	Zolmitriptan	4.597E-03	7.485E-04	8.165E-10	2.567E-03	361141	1.044E-04		
rs573907361	A	G	Zolmitriptan	3.776E-03	6.874E-04	3.955E-08	3.044E-03	361141	8.354E-05		
rs60236492	T	C	Zolmitriptan	4.907E-02	6.906E-03	1.205E-12	2.769E-05	361141	1.398E-04		
rs61752524	T	C	Zolmitriptan	3.061E-02	5.512E-03	2.814E-08	7.878E-05	361141	8.537E-05		
rs62001902	G	A	Zolmitriptan	7.342E-03	1.258E-03	5.326E-09	8.362E-04	361141	9.432E-05		
rs72756579	A	G	Zolmitriptan	2.350E-03	4.194E-04	2.100E-08	8.555E-03	361141	8.694E-05		
rs73208137	A	G	Zolmitriptan	2.425E-03	4.006E-04	1.416E-09	9.734E-03	361141	1.015E-04		
rs75994164	G	A	Zolmitriptan	3.754E-03	6.702E-04	2.140E-08	3.038E-03	361141	8.684E-05		
rs77102443	T	C	Zolmitriptan	1.164E-02	2.012E-03	7.247E-09	3.267E-04	361141	9.267E-05		
rs77412812	T	C	Zolmitriptan	3.287E-03	6.019E-04	4.737E-08	3.940E-03	361141	8.257E-05		
rs80351552	T	C	Zolmitriptan	1.610E-02	2.856E-03	1.739E-08	1.622E-04	361141	8.796E-05		

Abbreviations: SNP: single nucleotide polymorphism.

$$R\uparrow 2 =(2\times eaf\times(1-eaf)\times beta2)/[(2\times eaf\times(1-eaf)\times beta2)+(2\times eaf\times(1-eaf)\times N\times se2)]$$

$$F=N-k-1/k\times R\uparrow 2\ /$$
$$1-R\uparrow 2$$
$$N=4/(1/ncase+1/ncontrol)$$

The R^2 sum indicates the fraction of exposures elucidated by instrumental variables (IVs) across each pair of exposure and outcome.

N: effective sample size; k: number of IVs; eaf: effect allele frequency.

Table S3. Results of the LDSC and COLOC analyses.

Exposure	Outcome	LD Score Regression Analysis			Bayesian Colocalization Analysis					
		rg	rg_se	rg_p	nsnps	PP.H0.abf	PP.H1.abf	PP.H2.abf	PP.H3.abf	PP.H4.abf
Pregabalin	All anxiety disorders	0.206	0.115	0.074	6290	0.702	0.079	0.192	0.022	0.005
Bumetanide	All anxiety disorders	-	-	-	5250	0.753	0.066	0.162	0.014	0.004
Prednisolone	All anxiety disorders	0.076	0.085	0.369	15605	0.000	0.445	0.000	0.530	0.025
Vitamin b1 preparation	Dementia	-0.118	0.947	0.901	4319	0.148	0.275	0.195	0.362	0.020
Creon e/c granules in capsule	Epilepsy	-	-	-	6946	0.519	0.118	0.288	0.065	0.010
Pentasa sr 250mg m/r tablet	Multiple Sclerosis	0.613	0.378	0.105	3535	0.027	0.734	0.006	0.172	0.060
Zolmitriptan	Stroke, excluding SAH	-0.549	0.278	0.049	4903	0.744	0.075	0.161	0.016	0.005

Tip: "-" indicates a negative heritability estimate for the exposure trait in LDSC analysis.
